# When Fungi Meet Bacteria: Cross-Kingdom Assembly and Bioremediation Potential Under PAH Stress

**DOI:** 10.3390/jof12070469

**Published:** 2026-06-25

**Authors:** Anna Poli, Andrea L. Marchitelli, Irene Stefanini, Marina Bambi, Francesco Giunchino, Paola Calza, Giovanna Cristina Varese, Valeria Prigione

**Affiliations:** 1 Department of Life Sciences and Systems Biology, University of Torino, Viale Mattioli 25, 10125 Torino, Italy; anna.poli@unito.it (A.P.);; 2 National Biodiversity Future Center—NBFC, Piazza Marina 61, 90133 Palermo, Italy; 3 Department of Chemistry, University of Torino, Via Pietro Giuria 7, 10125 Torino, Italy

**Keywords:** PAH biodegradation, fungal–bacterial interactions, metabarcoding, culturomics, urban soil remediation

## Abstract

Polycyclic aromatic hydrocarbons (PAHs) are persistent and toxic pollutants that accumulate in urban soils, reducing microbial diversity and compromising ecosystem functioning. Developing effective bioremediation strategies requires identifying native degraders and understanding their ecological dynamics under pollutant pressure. Here, we investigated fungal and bacterial communities from PAH-contaminated soil subjected to three consecutive enrichment steps using phenanthrene, fluoranthene, benzo(a)pyrene, benzo(g,h,i)perylene, and their mixture as the sole carbon sources. High-throughput sequencing of ITS2 and V3-V4 amplicons revealed a decline in alpha diversity and a strong restructuring of both communities during the enrichment. Distance-based redundancy analysis showed that contaminant type and enrichment progression jointly shaped community composition, selecting for stress-tolerant taxa. Culturomics yielded 102 fungal isolates, representing 19 taxa, predominantly within Ascomycota. The most represented taxa were *Galactomyces pseudocandidus* (19 strains), *Fusarium oxysporum* (five), *Stilbella aciculosa* and *Exophiala attenuata* (four each) and *Fusarium solani* (three). Approximately one-third of isolates harbored associated bacteria, mainly *Stenotrophomonas*, *Bosea* and *Chitinophaga* species. Functional assays identified biosurfactant-producing strains, while microplate screening highlighted *Fusarium solani*, *Galactomyces pseudocandidus* and *Trametes versicolor* as capable of growing under PAH-selective conditions. Overall, our results demonstrate that PAH-contaminated soils host fungal taxa able to persist under pollutant pressure together with recurrent fungi-associated bacteria of potential ecological relevance for bioremediation.

## 1. Introduction

Soil is a dynamic and complex ecosystem that, directly or indirectly, supports the life of all organisms on Earth. Indeed, this environmental matrix is home to a variety of microorganisms (including archaea, bacteria and fungi) that, with their reciprocal interactions, provide regulating and supporting ecosystem services such as pest control, nutrient cycling, plant growth promotion and many others [1,2,3]. However, soil quality is continuously threatened by several anthropogenic activities (e.g., increase in industrial activities, combustion of fossil fuels and organic matter, traffic pollution, etc.) that result in an increasing accumulation of polycyclic aromatic hydrocarbons (PAHs), polychlorinated biphenyls (PCBs) and pesticides [4,5,6,7,8]. Like other xenobiotics, PAHs are persistent in urban soils and, due to their carcinogenic/mutagenic effects, negatively affect environmental and animal health [5,8]. Indeed, consisting of two or more aromatic benzene rings fused and arranged in linear or complex structures, these ubiquitous pollutants are highly recalcitrant [5,9,10]. Therefore, contamination of soil is a serious environmental issue that requires original, feasible, and sustainable solutions. While physicochemical methods are time-consuming and costly [8,11], nature-based solutions (phyto- and bioremediation) have been regarded as effective and eco-friendly approaches that rely on the use of microorganisms and plants to restore a contaminated site [12,13,14]. Specifically, bioremediation harnesses microbial metabolism to stabilize or convert contaminants into less toxic compounds [15]; resident microorganisms adapted to polluted environments can, through specific enzyme systems, leverage xenobiotics such as PAHs for their own growth [15,16]. Fungi are widely accepted as degraders of recalcitrant organic compounds, being endowed with ligninolytic enzymes and cytochrome P450 monooxygenases mainly involved in the breakage of the aromatic structures [14,17,18]. In a PAH-contaminated soil, the expression of ligninolytic enzymes (i.e., lignin peroxidase, Mn-peroxidase, laccase and versatile peroxidase) is triggered by the presence of both organic pollutants and lignocellulosic substrates such as plant debris, which could act as natural inducers of this enzymatic arsenal, thus enhancing PAH mineralization via co-metabolism [19]. However, several challenges hamper this natural degradative potential: first, when a soil is heavily contaminated, the development of an actively degrading microbial community is severely challenged; second, allochthonous microorganisms whose efficacy has been demonstrated *in vitro* may not survive in such harsh environments because of pollutant pressure and competition with autochthonous populations [20]. Bearing this in mind, either biostimulation (i.e., the boosting of resident active microorganisms’ growth) or bioaugmentation (i.e., the introduction of indigenous active microorganisms previously enriched and selected under laboratory conditions), depending on the type and/or degree of pollution [21], can be used to achieve a successful bioremediation. The efficacy of bioaugmentation may depend on the procedure of microbial enrichment in laboratory settings [22]. To this end, to isolate desired microorganisms in pure culture, it is pivotal to perform an enrichment strategy consisting of the use of target pollutants as the sole carbon source [23]. Another factor to consider is the extent to which contaminants are accessible for microbial degradation (their bioavailability), which can be modulated and increased through treatments of the soil with biosurfactants [24]. These amphipathic molecules, produced by microorganisms like fungi and bacteria, increase the mobility, availability and degradation of insoluble compounds like hydrocarbons [25,26]. Recent advances in bioremediation have highlighted carrier-assisted immobilization and microbial consortia as promising strategies to improve pollutant removal in contaminated soils. Biochar-, alginate- and nanomaterial-based carriers may enhance microbial survival, enzymatic stability and pollutant bioavailability [27], while microbial consortia may support degradation through complementary catabolic pathways, cross-feeding, biofilm formation and quorum sensing-mediated regulation [28]. Although not experimentally addressed here, these approaches provide an applied framework for future development of the native fungal–bacterial associations selected under PAH pressure.

In this study, we aimed to isolate fungal strains capable of growing on PAHs—benzo(a)pyrene, benzo(g,h,i)perylene, fluoranthene, and phenanthrene—provided as sole carbon sources using enrichment techniques applied to a contaminated urban soil. The isolated strains were identified and characterized for biosurfactant production. In parallel, metabarcoding analyses were performed to track shifts in fungal and bacterial communities throughout the enrichment process. By combining these approaches, we investigated the ecological dynamics of native microbial populations under PAH-selective conditions and explored potential inter- and intra-kingdom interactions relevant to bioremediation.

## 2. Materials and Methods

### 2.1. Sampling Site and Collection

Soil samples were collected from an 83 m^2^ plot within Meisino Park (Torino, Italy; 45°5′29.935″ N, 7°43′21.585″ E), a peri-urban natural area of approximately 45 ha comprising open fields and woodlands at the confluence of the Po and Stura di Lanzo rivers. Part of the park previously hosted 54 urban gardens arranged in fenced sections and corridors. The site was used for horticulture until 2020, when the regional Environmental Protection Agency (ARPA Piemonte) detected contamination by PAHs and heavy metals. Although the exact sources of pollution remain uncertain, potential contributors include industrial activities, traffic from adjacent urban areas, and historical horticultural practices involving combustion residues and agrochemicals [29]. According to the measured particle-size distribution, the soil was classified as sandy/loam [30]. Approximately 500 g of topsoil were sampled at a depth of 10–15 cm below the lawn surface from six distinct points located three meters apart. Samples were placed in sterile plastic bags and transported to the laboratory; a representative composite sample was obtained by pooling and homogenizing the six samples (100 g each), which was then sieved through a 2 mm mesh to remove stone and plant debris and stored at 4 °C until analysis.

### 2.2. Target PAHs

The soil investigated showed high concentrations of phenanthrene (PHE, 113 μg Kg^−1^ dw), fluoranthene (FLUO, 162 μg Kg^−1^ dw), benzo(a)pyrene (BaP, 115 μg Kg^−1^ dw) and benzo(g,h,i)perylene (BghiP, 103 μg Kg^−1^ dw) [30]; accordingly, these contaminants were chosen as representative of different classes of PAHs. In addition, the microbial enrichment was performed in a setting including a mixture of the four contaminants. All chemicals were purchased from Merck (Darmstadt, Germany). PAH stock solutions were prepared in 100% acetone. Before inoculation, PAH-amended Erlenmeyer flasks were kept under a chemical hood overnight to allow most of the acetone to evaporate. Final concentrations were 200 ppm FLUO and PHE, 50 ppm BaP, and 20 ppm BghiP; MIX consisted of 50 ppm FLUO, 50 ppm PHE, 25 ppm BaP and 5 ppm BghiP. These concentrations were chosen to impose a strong selective pressure while maintaining conditions compatible with fungal survival and growth. Lower concentrations were used for high molecular weight PAHs, particularly BaP and BghiP, because of their lower solubility and higher recalcitrance/toxicity compared with PHE and FLUO.

### 2.3. Enrichment in Liquid Culture and Fungal Isolation

Ten grams of fresh soil were added to 250 mL Erlenmeyer flasks containing 90 mL of sterile mineral medium (MM) and the target pollutant (or the mixture) as the sole carbon source. The MM was supplemented with antibiotics (Gentamicin 80 mg L^−1^ and Tazobactam 100 mg L^−1^) to prevent bacterial growth. The flasks were incubated for seven days on a rotary shaker at 180 rpm and 24 °C. Next, 5 mL of the culture, containing suspended biomass but avoiding coarse soil particles as much as possible, were transferred to a new flask containing fresh MM and the corresponding PAH. MM consisted of: NaNO_3_ 2 g L^−1^, KH_2_PO_4_ 1 g L^−1^, NH_4_Cl 1 g L^−1^, mineral solution (MS; KCl 50 g L^−1^, MgSO_4_·7H_2_O 50 g L^−1^, FeSO_4_·7H_2_O 1 g L^−1^) 10 mL L^−1^, and trace metal solution (TMS; ZnSO_4_·7H_2_O 10 g L^−1^, CuSO_4_ 5 g L^−1^) 1 mL L^−1^. Three consecutive enrichment steps were performed. At the end of each step, the biomass was harvested by filtration through a sterile cheesecloth for environmental DNA extraction as described in Section 2.5. Each condition was performed in triplicate.

A total of 1 mL of the last enriched culture was diluted in 0.9% NaCl (1:20) and plated onto 15 cm Petri dishes filled with agarized MM supplemented with antibiotics (Gentamicin 80 mg L^−1^ and Tazobactam 100 mg L^−1^) and the corresponding PAHs (individual or mixture) as the sole carbon source. The concentration of pollutants was the same as for the liquid cultures. For each flask, 5 plates were inoculated, ending up with a total of 75 plates (5 plates × 5 treatments × 3 replicates). Plates were incubated at 24 °C until the complete development of fungal colonies, which were transferred to malt extract agar (MEA: 20 g L^−1^ malt extract, 20 g L^−1^ glucose, 2 g L^−1^ peptone, 20 g L^−1^ agar) to obtain pure cultures.

As the enrichment procedure was specifically designed to select fungi, bacteria were not isolated or functionally screened as independent PAH degraders in the present study. The bacterial fraction detected after enrichment should therefore be interpreted as the component able to persist under the combined selective pressure of PAHs and antibiotics.

### 2.4. Chemical Analyses

To assess the progressive pollutant depletion, we performed chemical analyses at the beginning and at the end of the three successive steps of the liquid enrichment on PHE (see Section 2.3).

Briefly, 5 mL of sample was filtered through a 0.22 µm hydrophilic cellulose syringe filter, diluted 5 times in acetonitrile (ACN) and re-filtered through 0.45 HDPE filters. Samples were analyzed for phenanthrene content with an Agilent™ HPLC-UV-FL instrument (Agilent, Waldbronn, Germany), with modules belonging to the 1100/1200 series, and equipped with a Restek™ Pinnacle II PAH (Restek Corporation, Bellefonte, PA, USA) (150 mm × 4.6 mm, 4 µm) column fitted with a precolumn. Chromatographic conditions: isocratic elution with H_2_O/ACN mixture (30/70), flow rate 1 mL/min, runtime 5 min, UV λ = 260 nm, FL λ (ex) = 259 nm, λ (em) = 365 nm, oven T = 25 °C. LC-grade acetonitrile was purchased from Sigma-Aldrich™, ultrapure water was produced by a MilliQ™ EQ7000 system (Millipore SAS, Molsheim, France).

### 2.5. eDNA Extraction and Metabarcoding

Environmental DNA was extracted from about 0.25 g of each biomass at the end of every enrichment step by following the manufacturer’s instructions of a DNeasy PowerSoil Pro Kit (Qiagen, Carlsbad, CA, USA). Quantity and quality were checked as in Section 2.7. A metabarcoding analysis was applied to track the dynamic shift of both fungal and bacterial communities during the enrichment progression, resulting in three time points. While the fungal community reflected taxa selected under PAH pressure, the bacterial community represented taxa able to persist under the combined selective pressure of PAHs and the antibiotics used during the enrichment procedure. Environmental DNA extracted directly from soil served as a control (CTRL, corresponding to T0).

For fungi, the target region was amplified through a semi-nested PCR approach. In the first PCR, the nrITS (ITS1-5.8S-ITS2) region was amplified by using the primer pair ITS1F/ITS4 [31]. For the second PCR, primers fITS9/ITS4 [32] coupled to Illumina overhang adapters were used to amplify the ITS2 region. For bacteria, the 16S V3-V4 regions were amplified by using primers 27F/1492R for the first PCR and S-D-Bact-0341-b-S-17 and S-D-Bact-0785-a-A-21 [33] coupled to Illumina overhang adapters for the second amplification.

For a total volume of 25 µL, reactions consisted of 1 µL DNA, 10 × PCR Buffer (15 mM MgCl_2_, 500 mM KCl, 100 mM Tris-HCl, pH 8.3), 200 µM each dNTP, 1 µM each primer, and 2.5 U Taq DNA Polymerase (Qiagen, Chatsworth, CA, USA). Negative controls were included. Amplifications were performed in triplicate. Amplicons were visualized on a 1.5% agarose gel stained with SYBR™ Safe (Thermo Fisher Scientific, Waltham, MA, USA) together with a GelPilot 1 kb plus DNA Ladder. The three replicates per sample were pooled, purified using the Wizard SV Gel and PCR Clean-Up System (Promega, Milano, Italy), and quantified with a Qubit 2.0 (Invitrogen, Carlsbad, CA, USA). Paired-end sequencing (2 × 300 bp) using the Illumina MiSeq platform was performed at IGA Technologies (Udine, Italy).

### 2.6. Bioinformatic Analysis

Fungal demultiplexed ITS2 sequences were processed using QIIME2 (Quantitative Insights Into Microbial Ecology 2) version 2024.2 [34]. Following removal of adapters and primers from fungal and bacterial raw data, the DADA2 algorithm was used for denoising and quality control, including chimera removal and trimming [34,35]. Amplicon sequence variants (ASVs) were identified; singletons and rare ASVs were discarded (<10). Fungal taxonomy was assigned using the UNITE QIIME classifier release for Fungi (version 9.0—16.10.2022). Prokaryotic taxonomy was assigned using the SILVA database v.138.1 [36]. Raw sequencing data are available in the NCBI Sequence Read Archive (SRA) under accession No. PRJNA1371905.

### 2.7. Identification of Isolates—DNA Extraction, PCR Amplification and Sequence Assembly

To obtain genomic DNA of the isolates in pure culture, approximately 100 mg of fresh mycelium were scraped from MEA plates and transferred to a 2 mL Eppendorf tube prior to disruption by means of a MM400 tissue lyzer (Retsch GmbH, Haan, Germany); thereafter, the manufacturer’s instructions of a NucleoSpin Plant II Kit (Macherey Nagel GmbH, Duren, DE, USA) were followed. The quality and quantity of DNA were measured spectrophotometrically (Infinite 200 PRO NanoQuant; Tecan, Männedorf, Switzerland) and samples were then stored at −20 °C until use.

PCR amplification of specific markers was performed in a T100 Thermal Cycler (Bio-Rad, Hercules, CA, USA). Primer pairs ITS1/ITS4 [31] and LR0R/ LR7 [37] were used to amplify the internal transcribed spacer, including the 5.8S rDNA gene (nrITS) and the 28S large ribosomal subunit (nrLSU), respectively. The β-tubulin (β-tub; for the genera *Aspergillus* and *Penicillium*) and the α-actin (α-act; for the genus *Cladosporium*) genes were amplified using primer pairs Bt2a/Bt2b [38] and ACT512F/ACT783R48 [39], respectively; primers EF1/EF2 [40] served to amplify the translation elongation factor 1α (TEF; for the genus *Fusarium*). To assess potential fungal–bacterial associations, primers 27F/1492R were used to amplify the bacterial 16S ribosomal region [41]. The reaction mixture consisted of 40–80 ng DNA template, 10 × PCR Buffer (15 mM MgCl_2_, 500 mM KCl, 100 mM Tris-HCl, pH 8.7), 200 µM each dNTP, 1 µM each primer, and 2.5 U Taq DNA Polymerase (Qiagen, Chatsworth, CA, USA) in a total volume of 50 µL. Negative controls with no DNA template were included. The thermocycler was programmed as previously described [42,43].

PCR products were checked on an agarose gel as described in Section 2.5, purified and Sanger sequenced at the Macrogen Europe Laboratory (Milano, Italy). The software Sequencer 5.2 (GeneCodes Corporation, Ann Arbor, MI, USA, http://www.genecodes.com, accessed on 20 June 2026) was used to inspect, trim and assemble the resulting Applied Biosystem (ABI) chromatograms in consensus sequences. Newly generated sequences were compared to those available in public databases (GenBank—nblast; mismatch 1/−2; gap costs linear; Mycobank) and deposited at NCBI (**ITS**: PX519207–PX519255; **LSU**: PX363221–PX363240, PX443406, PX443407; **α-act**: PX312444–PX312447; **β-tub**: PX312442, PX312443; **TEF**: PX312448–PX312454). A sequence similarity of ≥98% (e value > e^−100^) was considered reliable; results were confirmed morphologically.

### 2.8. PCR Fingerprinting

Fungal isolates belonging to the same species were subjected to dereplication (based on the amplification of hypervariable repetitive DNA sequences within fungal genomes) to determine whether or not they corresponded to the same strain. Primers for the minisatellite-specific core sequence of the wild-type phage M13 (5′-GAGGGTGGCGGTTCT-3′) and the microsatellite-specific sequences (GTG)_5_ and (GACA)_4_ were used as single primers [44,45]. PCR amplification was carried out in a final volume of 25 μL containing 25 ng DNA, 10 × CoralLoad PCR Buffer (15 mM MgCl_2_, 500 mM KCl, 100 mM Tris-HCl, gel loading reagent, orange dye, red dye; pH 8.7), 200 µM each dNTP, 1.2 µM primer and 2.5 U Taq DNA Polymerase (Qiagen, Chatsworth, CA, USA). A T100™ thermal cycler (Bio-Rad) was programmed as follows: 95 °C for 5 min, 40 × (93 °C for 45 s, 50 °C for 1 min, 72 °C for 1 min), 72 °C for 6 min. The amplified products were resolved by electrophoresis on a 1.5% agarose gel in TBE buffer for 3 h at 35 V. The resulting fingerprints were visualized under UV light using a Gel Doc™ XR system (Bio-Rad) equipped with image analysis software.

Representative strains of each species isolated in this work are preserved at the *Mycotheca Universitatis Taurinensis* (MUT—https://www.tucc-database.unito.it/mut, accessed on 20 June 2026).

### 2.9. Biosurfactant Production

Fungal strains were evaluated for their ability to produce biosurfactants. Agar plugs from pre-grown fungal cultures were inoculated in 50 mL Erlenmeyer flasks containing 30 mL of modified mineral salt medium (MSM; KH_2_PO_4_ 0.3 g L^−1^, MgSO_4_ 0.3 g L^−1^, NaNO_3_ 3 g L^−1^, yeast extract 2 g L^−1^, soybean oil 40 mL g L^−1^ and glucose 5 g L^−1^) to induce the production of surfactants. The flasks were incubated at 24 °C and 120 rpm for 7 days. To remove the mycelium, cultures were first filtered through a cheesecloth; the filtered liquid was then centrifuged at 7000× *g* for 30 min at 4 °C. Biosurfactant production was qualitatively assessed using three assays: (i) the drop collapsing assay; (ii) the oil spreading test; and (iii) the emulsification test. Tween-80 (pure and 1% *v*/*v* solution) and distilled H_2_O were used as positive and negative controls, respectively.

(i) Drop collapsing assay (DCA)

A total of 5 μL of paraffin oil was placed onto the lid of a 96-well microplate, as described by Bodour et al. [46]. The oil-coated wells were equilibrated for two hours at room temperature before adding 5 µL of the cell-free supernatant. After 1 min, droplet shape was visually evaluated; spreading indicated a positive result, whereas a stable beaded drop indicated the absence of biosurfactant [47]. The test was performed in triplicate.

(ii) Oil displacing test (ODT)

The ODT was performed following the method described by Morikawa et al. [48]. Briefly, 40 mL of distilled H_2_O were poured into a 15 cm diameter Petri dish. Then, 40 µL of crude oil were carefully added to the water surface, followed by 20 µL of culture supernatant applied at the center of the oil layer. After a few seconds, in the presence of biosurfactants, the oil is displaced, forming a visible clearing zone [49].

(iii) Emulsification activity (EI)

To evaluate emulsification activity, a 2 mL aliquot of olive oil was mixed with 2 mL of cell-free supernatant in a screw-cap tube. The mixture was vortexed vigorously for 2 min and left at room temperature. The EI was calculated after 24, 48 and 72 h by dividing the height of the emulsion layer by the total height of the mixture and multiplying by 100. The EI values at different time points serve as indicators of emulsion stability [47,50].

### 2.10. Microplates Screening

A high-throughput screening in 96-well microplates was used to evaluate the ability of the individual strains to grow in the presence of each target pollutant and the mixture.

Fungi were pre-cultured on MM plates at 24 °C. Inoculation was carried out using the microplug method as described by McNab et al. [51]. Briefly, a 1000 µL pipette tip was pressed perpendicularly onto the fungal culture to extract a plug, which was then transferred into a microplate well. Next, 200 μL of MM supplemented with each pollutant (previously prepared in 250 mL Erlenmeyer flasks) were dispensed in each well.

Several types of controls were included in the experimental design. Inoculum in MM supplemented with glucose 20 g L^−1^ served as a quality control; absence of fungal growth in the presence of glucose invalidated the experiment. Negative controls consisted of MM and empty microplugs, while MM supplemented with acetone was used to assess possible solvent-related effects, since acetone was used to dissolve PAHs. Each condition was tested with eight replicates, each paired with its corresponding blank (empty microplug). To minimize evaporation, peripheral wells were filled with 200 µL of sterile deionized water. Microplates were incubated at 24 °C in the dark; growth was monitored by measuring absorbance at 405, 595, and 630 nm [51] using the plate reader infinite M2000 with Magellan V 6.5 software (TECAN, Grödig, Austria, 10 flashes/measurement). Measurements were recorded at 0, 24, 48, 72, 168, 240, 336 and 504 h.

### 2.11. Statistical Analyses

After the removal of one low-depth sample (<1312 reads, one of the fungal control samples), alpha diversities (Observed richness, Chao1, and Shannon) were computed with the phyloseq package, and differences across steps (0–3) and treatments were evaluated by Analysis of Variance (ANOVA) with Tukey’s HSD post hoc test.

Beta diversity was assessed with the Bray–Curtis dissimilarity index on TSS-normalized data (total-sum scaling to relative abundances) and analyzed via distance-based RDA (dbRDA) to quantify variation attributable to the treatment (i.e., type of PAH) and to the step of the enrichment procedure; significance was tested by permutation ANOVA (999 permutations) in vegan. Differences among treatments were tested by Permutational Multivariate Analysis of Variance (PERMANOVA) implemented in vegan’s adonis2 function. Pairwise contrasts were conducted with the pairwiseAdonis package. We used Wilcoxon tests on Bray–Curtis distances to assess treatment and step effects, with False Discovery Rate (FDR) correction, by comparing intra-group dispersion against inter-group distance. Differential taxa abundance across treatments and steps was assessed with the run_LEfSe function from the microbiome Marker package with an LDA threshold of 2.0. Cross-kingdom associations were evaluated with Spearman’s rank correlation, with FDR correction across tests. All the analyses were performed in R (run in RStudio 2024.09.0+375).

## 3. Results and Discussion

### 3.1. Changes in Fungal and Bacterial Community Compositions Across Enrichment with Different PAHs

To evaluate microbial selection dynamics throughout the enrichment process, a metabarcoding analysis was performed. This approach allowed us to monitor the evolution of fungal and bacterial communities and to identify the taxa most actively involved in the degradation of the different PAHs.

**Figure 1:**
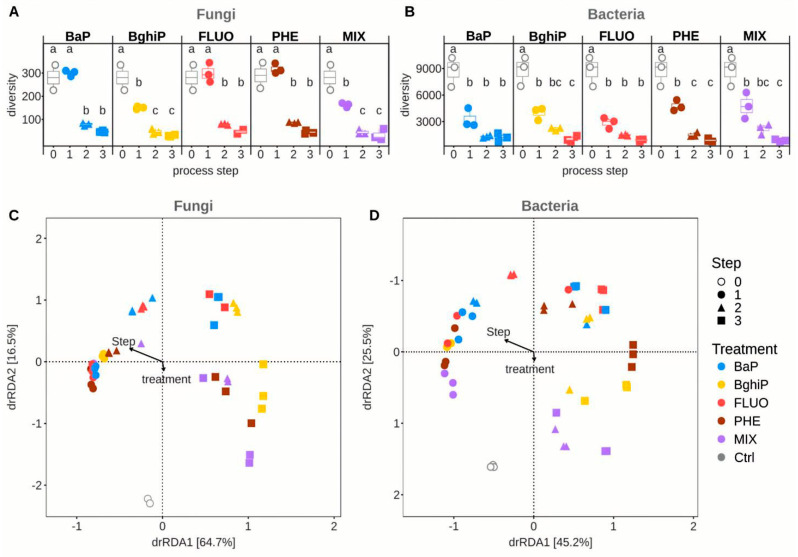
Comparison of fungal and bacterial diversity during the enrichment processes. (**A**) Fungal Observed alpha diversity (number of fungal taxa observed in various samples); (**B**) bacterial Observed alpha diversity (number of bacterial taxa observed in various samples). In panels (**A**,**B**), different lowercase letters indicate significant differences among groups (Wilcoxon–Mann–Whitney FDR < 0.05). (**C**) dbRDA analysis of Bray–Curtis fungal community diversity; (**D**) dbRDA analysis of Bray–Curtis bacterial community diversity.

The ITS2 dataset generated 5,435,379 forward and reverse reads, resulting in 4,511,047 quality-filtered fragments. Following denoising and chimera filtering, 3,248,659 reads were obtained (ranging from 1318 to 189,078 reads per sample). Despite variation in sequencing depth among samples, the progressive flattening of the rarefaction curves (Figure S1) indicates that most of the diversity was detected. Alpha diversity of fungal and bacterial communities was assessed using Observed richness (Figure 1A,B), Chao1, and Shannon indices (Figure S2 and Table S1). A progressive decline in biodiversity was observed across all PAH treatments, including the mixture, as the enrichment proceeded (ANOVA; *p* < 0.05). In most cases, the Observed fungal alpha diversity index of the control sample did not differ significantly from those recorded after the first enrichment step. The only exceptions were BghiP and MIX, where a significant decrease occurred as early as the first enrichment step (post hoc Tukey’s HSD test *p* < 0.05; Figure 1A), indicating a rapid selection during the process. A further significant decrease was evident between the first and the second step, while no additional changes were detected between the second and the final step. For bacteria, significant reductions in Observed alpha diversity indices were detected in all treatments after the first enrichment step (post hoc Tukey’s HSD test, *p* < 0.05), with no further changes as the procedure progressed. PHE was the only exception; a significant decrease occurred between the first and the second step (post hoc Tukey’s HSD test, *p* < 0.05; Figure 1B). Shannon and Chao1 indices showed similar behaviors (Figure S2). These patterns are consistent with field and microcosm evidence showing that PAHs act as strong environmental filters (on both fungal and bacterial communities), with diversity reductions and network simplification at higher pollution levels [52,53].

The influence of PAH type and enrichment progression on fungal and bacterial community compositions was assessed using distance-based redundancy analysis (dbRDA; Bray–Curtis dissimilarities; Figure 1C,D). The first constrained axis (dbRDA1) explained 64.7% and 45.2% of the variation in fungal and bacterial communities, respectively, while the second axis (dbRDA2) accounted for an additional 16.5% and 22.5% (Figure 1C,D). In both communities, samples clustered primarily according to treatment (permutation ANOVA; *p* = 0.015; Table S2), and this effect intensified as the procedure advanced (permutation ANOVA; *p* = 0.003). At each enrichment step, both fungal and bacterial communities differed significantly from the control samples (i.e., soil prior to enrichment), indicating that selective processes affected both kingdoms from the very first enrichment cycle (Table S2). Late-stage convergence into a few treatment clusters is in line with network-level observations that high PAH burdens reduce complexity and stabilize specialized assemblages, with simplified fungal/bacterial co-occurrence networks at high contamination levels [52].

Ascomycota was the dominant phylum across all samples, ranging from 92% in the control to 100% in the third enrichment step of each treatment. Basidiomycota, which accounted for 3.7% in the control, decreased after the first enrichment step to ~1% with PHE, FLUO, and BaP, to 0.25% with MIX and to 0.14% with BghiP, whereas they reached 0.02% and 0.05% in the third enrichment step with BaP (BaP3) and MIX (MIX3), respectively. Mortierellomycota (2.79% in the control) remained detectable after the first step (0.07% with BghiP to 1.08% with PHE) and then declined. Minor phyla (Basidiobolomycota, Blastocladiomycota, Chytridiomycota, Mucoromycota, Olpidiomycota, Rozellomycota, Sanchytriomycota, and Zoopagomycota) and unidentified fungi together represented 0.74% in the control; they were still detected after the first step in each treatment but were no longer present in the second step with MIX (MIX2) and in the third step overall (Figure S3A). The strong rise in Ascomycota (up to ~100%) with a parallel decline in Basidiomycota is consistent with the culturomics results (see Section 3.3); a small set of stress-tolerant Ascomycota taxa dominate worldwide and often expand under severe perturbations, while Basidiomycota frequently lose dominance under harsh conditions [54]. At the functional level, enzyme profiling indicates broader substrate versatility in Ascomycota, while Basidiomycota generally excel in lignin oxidation. Notably, some white-rot Basidiomycota (e.g., *Trametes versicolor*) can contribute to HMW-PAH transformation via laccases and P450 systems, highlighting potential complementary roles depending on the treatment and step.

Within Ascomycota, Sordariomycetes was the most abundant class (85.70% in PHE1 to 98.40% in MIX3; control 40.65%), followed by Dothideomycetes (0.0041% in BghiP3 to 5.42% in PHE1; control 46.60%) and Eurotiomycetes (0.049% in MIX3 to 6.69% in PHE1; control 4.85%) (Figure S3B). Hypocreales dominated at the order level (90.65% in FLUO1 to 99.85% in MIX3; control 35.09%), with Sordariales, Glomerellales and Chaetothyriales present at much lower relative abundances (Figure S3C). Within the few Basidiomycota detected, Agaricomycetes and Tremellomycetes were the most abundant classes, with Agaricales and Tremellales the best-represented orders.

Focusing on Nectriaceae (the most abundant family within Hypocreales), marked shifts at the genus level were observed between the first and the last enrichment steps. In PHE1, the most abundant genera were *Fusarium* (86.17%), *Thelonectria* (4.83%) and *Rectifusarium* (3.38%), whereas in PHE3 *Fusicolla* (67.88%), *Fusarium* (15.46%) and *Paracremonium* (11.64%) prevailed. Similarly, *Fusarium* dominated in FLUO1 (76.88%) but decreased to 14.00% in FLUO3, where *Paracremonium* emerged (59.70%). With BaP, *Fusarium* decreased from 82.24% in BaP1 to 13.39% in BaP3, where *Paracremonium* predominated (62.32%). Under BghiP, *Fusarium* dropped from 74.35% to 0.22% by the third step, which was dominated by *Fusicolla* (67.03%). In MIX, *Fusarium* accounted for 91.02% of Nectriaceae in Step 1 and 9.08% in Step 3, while *Fusicolla* increased to 81.09%.

**Figure 2:**
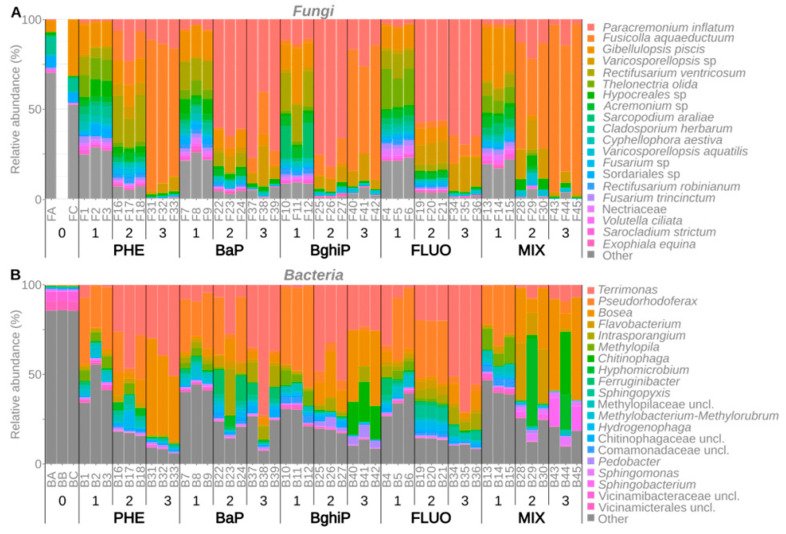
Bar plots of relative abundances of fungal species and bacterial genera in the enrichment steps. The relative abundances of the twenty most abundant fungal species (**A**) and bacterial genera (**B**) are shown for each analyzed sample, including enrichments with different PAHs (BaP, PHE, BghiP, FLUO, and mixture) and different steps of the process (1, 2, and 3), with the initial soil sample (0) as the control.

Overall, the most represented fungal species were *Paracremonium inflatum* (12.4%), followed by *Fusicolla aquaeductuum* (10.3%), *Gibellulopsis piscis* (4.14%), *Varicosporellopsis* sp. (2.97%), and *Rectifusarium ventricosum* (2.79%) (Figure 2A). The most represented bacterial genera were *Terrimonas* (10.33%), *Pseudorhodoferax* (8.74%), *Bosea* (6.1%), *Flavobacterium* (1.8%), *Intrasporangium* (1.51%), and *Methylopila* (1.41%) (Figure 2B). The fungal and bacterial communities were impacted by the treatment, with a clear shift already after the first enrichment step (Figure 2). The enrichment of bacterial genera such as *Terrimonas*, *Mesorhizobium*, and *Bosea* alongside selected Hypocreales is consistent with bioremediation systems where anthracene/phenanthrene amendments link these taxa to active degradation [55,56].

**Figure 3:**
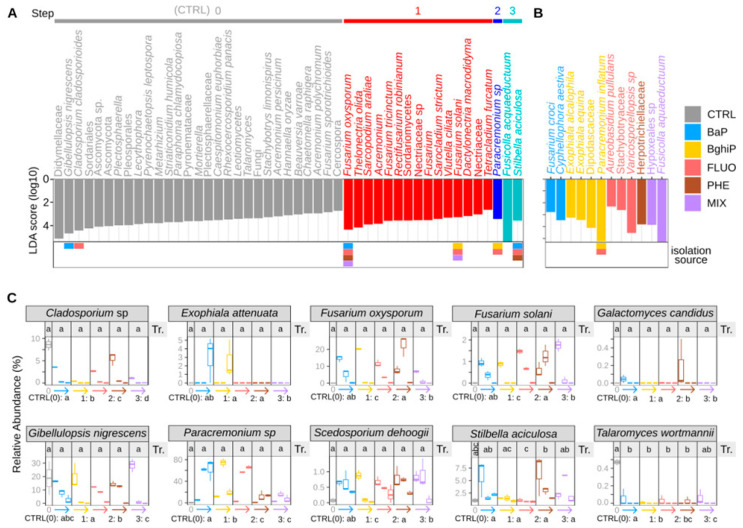
LEfSe results and dynamics of isolated fungal species. (**A**) Results of LEfSe analysis showing the fungal taxa characterizing the enrichment step indicated according to the bar color. The boxes on the left show the enrichment conditions, as reported in the legend, that allowed the isolation of strains belonging to the taxon. (**B**) Results of LEfSe analysis showing the fungal taxa characterizing the different enrichments. (**C**) Boxplots showing the relative abundances of the fungal species isolated at the end of the enrichment and identified in metabarcoding analysis. For each taxon, the letters indicated on the top of the boxplot indicate treatments with significantly different abundances of the taxon; the letters at the bottom of each boxplot show enrichment steps with significantly different abundances of the taxon (Wilcoxon–Mann–Whitney test FDR < 0.05). Colored boxes underneath the taxon names indicate the enrichment from which the species was isolated.

Linear discriminant effect size (LEfSe) analysis identified 29 fungal species significantly affected by the enrichment conditions (Figure 3A). Briefly, Step 1 was associated with 15 species, including *Fusarium oxysporum* and *Fusarium solani*, which were also isolated at the end of the process. Later steps were characterized by fewer taxa—*Paracremonium* sp. at Step 2 and *Fusicolla aquaeductuum* together with *Stilbella aciculosa* at Step 3 (Figure 3A)—consistent with progressive, treatment-specific impacts. Different selection patterns for each PAH treatment were also supported: *Fusarium croci* and *Cyphellophora aestiva* in BaP; *Exophiala alcalophila*, *Exophiala equina*, *Dipodascaceae*, and *Paracremonium inflatum* in BghiP; *Aureobasidium pullulans*, Stachybotryaceae, and *Varicosporellopsis* in FLUO; Herpotrichiellaceae in PHE; and *Hypocreales* sp. and *Fusicolla aquaeductuum* in the MIX (Figure 3B). These findings are consistent with the observation by Shen et al. [57], who reported PAH load restructuring both bacterial and fungal taxa. However, while LEfSe highlights differentially abundant taxa, complementary functional assays are required to confirm their direct contributions to PAH transformation rather than passive co-selection [58].

### 3.2. Co-Occurrences Between Fungi and Prokaryotes Across PAH Enrichments

**Figure 4:**
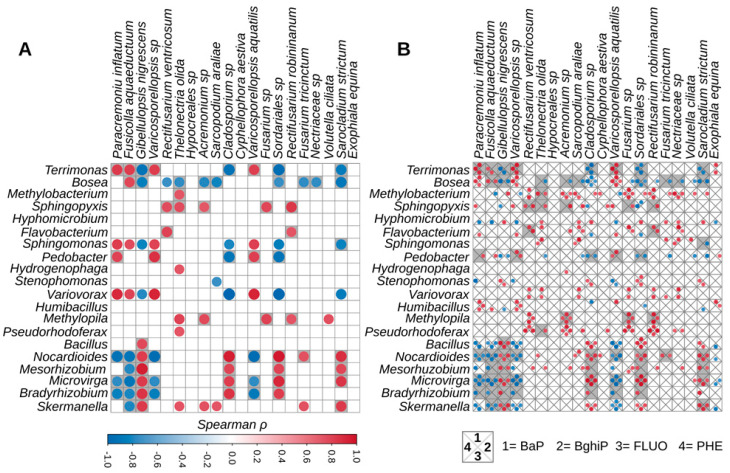
Correlations between fungal and bacterial taxa in the enrichment conditions. Spearman correlation rho values calculated among fungal (in columns) and bacterial (in rows) taxa. Only significant correlations (*p* < 0.05) with |rho| > 0.75 in the MIX enrichment (**A**) and the other treatments (**B**) are shown. Cells with a grey background highlight correlations found in the MIX sample and in at least one of the other treatments (with BaP, BghiP, FLUO, or PHE).

To explore putative co-occurrences between fungi and prokaryotes across PAH enrichments, we computed Spearman correlations among relative abundances and retained only significant pairs (ρ > 0.75; *p* < 0.05). In a few cases, correlations were observed in the MIX and in the single-PAH treatment, suggesting potential interplays between these taxa in the presence of PAHs (Figure 4). *Gibellulopsis nigrescens* (isolated with the culturomic approach) showed several positive correlations, both in MIX (Figure 4A) and single-PAHs (Figure 4B), with bacterial genera known to have a role in PAH degradation: *Nocardioides*, producing the enzyme phenanthrene dioxygenase responsible for PHE breakdown [59], and *Mesorhizobium* and *Bradyrhizobium*, included in microbial communities of anthracene bioremediation sites [55]. The correlation between *G. nigrescens* and *Bradyrhizobium* was found only in the PHE enrichment, suggesting that the known potential of this bacterial genus in degrading BaP [60] can make the association weaker, as both taxa are capable of dealing individually with the environmental settings. Finally, the genus *Microvirga* correlated positively with *Cladosporium herbarum* and *Sordariales* sp. and negatively with *Paracremonium inflatum*, *Varicosporellopsis* sp., and *V. aquatilis* in both MIX and single-PAHs (Figure 4A,B). These correlations indicate co-occurrence patterns rather than direct interactions and should be interpreted cautiously. Interestingly, *Bosea* and *Variovorax* are among the genera found in association with strains of *Galactomyces pseudocandidus* and *T. versicolor*, respectively (Section 3.4), which indicates actual fungi–bacteria relationships.

Co-occurrence networks based on relative abundance data reveal associations rather than causality. In this context, the recurrent fungal–bacterial associations observed here may represent naturally selected consortial units, potentially supporting pollutant tolerance and transformation through spatial proximity, chemical signaling and metabolic complementarity, although these mechanisms were not directly tested in the present study. Nevertheless, the positive correlations observed between fungal taxa, such as *G. nigrescens*, and bacterial genera previously associated with PAH degradation suggest possible ecological links under pollutant pressure. For instance, *Nocardioides* has been reported as a phenanthrene-degrading genus [59], *Bradyrhizobium* includes strains capable of BaP degradation [60], and *Bosea* species are repeatedly enriched in PHE systems or mixed consortia [56]. Moreover, fungal–bacterial co-cultures often outperform monocultures on PAH mixtures, as fungal extracellular enzymes enhance substrate accessibility for bacterial ring-hydroxylating dioxygenases [61]. Therefore, these correlations may be interpreted as a need for metabolic complementarity in severely polluted conditions. Alternatively, or in addition, fungal–bacterial associations may provide ecological advantages to bacteria exposed to the selective pressures of both PAHs and antibiotics, for instance, through possible fungal-mediated attenuation of antibiotic stress or through the use of fungal hyphae as protected microhabitats, potentially including endohyphal bacterial lifestyles.

As analytical evidence of pollutant depletion at the community-enrichment level, PHE concentration was measured at the beginning and at the end of each enrichment step. These analyses showed that, in every cycle, approximately 25–55% of the initial PHE was no longer detected in the analyzed liquid fraction at the end of each enrichment cycle (Figure S4). Since adsorption onto biomass or particulate material cannot be excluded, these data should be interpreted as evidence of PHE depletion during enrichment rather than definitive proof of biodegradation.

### 3.3. Solid Screening: Fungal Isolation and Identification

Selecting fungi with specialized metabolic capabilities shaped by long-term exposure to xenobiotics represents a promising approach for bioremediation [8]. The final solid-phase enrichment on individual PAHs and their mixture successfully selected fungi capable of tolerating toxicity and growing under conditions in which the target pollutants were supplied as the only carbon source. With the exception of one Basidiomycota (*T. versicolor*), all the isolates belonged to Ascomycota, and were ascribable to 19 taxa (Table 1). The isolated taxa were affiliated with five classes, eight orders, nine families, and 12 genera. The dominance of Ascomycota is consistent with their widespread occurrence and stress tolerance in soil ecosystems [23,30,62].

The most represented classes were Sordariomycetes (eight taxa), Eurotiomycetes (five taxa) and Dothideomycetes (four taxa); Dipodascomycetes was the most frequently isolated class, with 59 isolates assigned to one taxon. Only one isolate belonged to Agaricomycetes (Table S2). The best-represented genera were *Cladosporium* (four species), followed by *Fusarium* (three species), *Penicillium* and *Scedosporium* (two species each). These genera are frequently reported in association with PAH degradation. For instance, *Fusarium* spp. oxidize BaP and enhance the degradation of mixed PAHs in co-cultures [63,64,65], *Cladosporium* isolates from marine and terrestrial matrices degrade anthracene and additional PAHs [66,67], likewise Eurotiales members (*Aspergillus, Talaromyces,* and *Penicillium* species) frequently detected in contaminated environments [68,69].

Fungal taxa were differentially recovered depending on the PAH treatment. Twelve species (*Aspergillus fumigatus*, *Cladosporium cladosporioides*, *Cladosporium westerdijkia*, *E. attenuata*, *F. oxysporum*, *F. solani*, *G. pseudocandidus*, *Paracremonium* sp., *Penicillium crustosum*, *S. aciculosa*, *T. versicolor* and *Talaromyces wortmannii*) were isolated from FLUO, six (*Cladosporium allicinum*, *E. attenuata*, *F. oxysporum*, *G. pseudocandidus*, *G. nigrescens* and *S. aciculosa*) from BaP, five (*Cladosporium langeronii*, *F. oxysporum*, *G. pseudocandidus*, *Penicillium chrysogenum* and *S. aciculosa*) from PHE, five (*Fusarium falciforme*, *F. oxysporum*, *Fusarium solani*, *G. pseudocandidus* and *Scedosporium dehogii*) from the mixture of the four PAHs, and four (*F. solani*, *G. pseudocandidus*, *Paracremonium* sp. and *Scedosporium apiospermum*) from BghiP. *G. pseudocandidus* was isolated from each contaminant, including the mixture, while *F. oxysporum* was recovered from all the conditions except BghiP. *F. solani* and *S. aciculosa* were retrieved from three conditions, while *E. attenuata* and *Paracremonium* sp. were isolated each from two target pollutants. The remaining 13 species were detected in one condition only (Table S3). Our observation that each condition yielded partly distinct assemblages may be consistent with the ring-number dependence. Indeed, HMW PAHs (BaP, BghiP) are strongly recalcitrant, which makes their effective degradation complex, requiring either white-rot systems or concerted intracellular activation, whereas LMW PAHs (PHE, FLUO) are more tractable and can be transformed by a broader number of Ascomycota [70].

Thirty-seven unique strains were obtained following dereplication of isolates of the same species. *G. pseudocandidus* resulted in the species with the highest number of strains (19) followed by *F. oxysporum* (five), *S. aciculosa* and *E. attenuata* (four strains each), *F. solani* (three) and *G. nigrescens* (two). Although direct evidence of PAH metabolism by *G. pseudocandidus* is limited, the genus is known for extracellular enzyme production and bioindustrial versatility, and together with related species (i.e., *Geotrichum candidum*) is frequently isolated from harsh matrices [71,72,73].

To place the culturomics results in a broader ecological context, we compared the taxa recovered in culture with the metabarcoding profiles obtained across enrichment steps and PAH treatments. This integrative approach allowed us to assess whether the isolates reflected only cultivable endpoints of the selection process or taxa that also persisted and became enriched in situ under pollutant pressure. Overall, metabarcoding corroborated the persistence of several fungal taxa isolated through culturomics, while also helping to clarify their treatment-specific dynamics and relative ecological relevance (Figure 3C). *E. attenuata*, recovered from BaP and FLUO enrichments, showed high relative abundance in BaP and BghiP at Step 3. The presence of *G. nigrescens* in BaP at Step 3 matched its isolation and further emerged among soil-characteristic taxa (Figure 3A). Isolation of *Paracremonium* sp. from BghiP and FLUO was supported by metabarcoding profiles which also showed notable representation in BaP. Likewise, the isolation of *S. dehoogii* from the MIX enrichment was corroborated by amplicon data, which also detected high relative abundance in BaP and PHE samples (Figure 3C). The relative abundance of *G. pseudocandidus* was variable, typically moderate and with late increases (Steps 2–3) in selected treatments, consistent with their frequent recovery in culture across conditions despite low-to-moderate representation in the amplicon profiles (Figure 3C). It should be noted that the correct identification of this species was challenging due to nomenclatural overlap across repositories (UNITE reports it as *G. candidus*) and was ultimately resolved through a thorough polyphasic approach including phylogenetic analysis.

### 3.4. Fungi–Bacteria Associations

In recent years, fungal–bacterial associations have been increasingly reported in contaminated soils, suggesting that these kingdoms often respond jointly to environmental stressors and may participate in coordinated degradation processes [74,75]. This co-occurrence was assessed by PCR amplification of the bacterial 16S rRNA region from each fungal genomic DNA. Amplification succeeded in 34 out of 102 isolates (33%), indicating a stable association. Sequence analysis allowed the identification at the species or genus level of 26 fungal-associated bacteria, while eight samples yielded reproducible double peaks in Sanger chromatograms, indicative of putative multispecies bacterial consortia (Table S3). Overall, after excluding nine isolates (seven lost cultures and two identified as the opportunistic pathogen *E. attenuata*), *Stenotrophomonas* spp. (12) were the most frequent bacteria, followed by *Bosea robiniae* (six) and *Chitinophaga* spp. (six); occasional associations involved *Variovorax boronicumulans* and *Pseudomonas* sp. (one each). The fungal species exhibiting the highest frequency of bacterial association was *G. pseudocandidus*. Among the 59 isolates of this taxon, a diverse array of bacterial partners was recovered, including *Stenotrophomonas* spp. (11), *Bosea robiniae* (five), *Chitinophaga ginsengisegetis* (two), *Variovorax boronicumulans* (one), and *Pseudomonas* sp. (one). Notably, different bacterial taxa were observed across isolates of the same fungal species, suggesting a variable and potentially environment-dependent recruitment of bacterial partners rather than a strict specific symbiosis.

The recurrent associations, especially within *G. pseudocandidus*, support the hypothesis that bacteria may reside inside or along fungal hyphae as surface-associated consortia that exploit hyphal networks as dispersal corridors in water-unsaturated soils [74,76]. In this context, endohyphal bacteria, often termed “hidden facilitators”, are increasingly recognized as modulators of fungal phenotypes and secondary metabolism [76]. Concurrently, “fungal highways” can mobilize pollutant-degrading bacteria across the soil, thereby increasing encounter rates with otherwise inaccessible contaminants and enhancing biodegradation [74,75,77]. Such interactions may enhance PAH degradation through complementary metabolic pathways, with fungi providing oxidative enzymes like laccases, peroxidases, P450 monooxygenases and bacteria complementing ring-cleavage and mineralization pathways [58,70,78]. Our findings corroborate the dual role of bacteria as endohyphal residents and hyphal-surface associates that participate in PAH transformation within fungi–bacteria consortia. In particular, the genus *Stenotrophomonas* is enriched on fungal hyphae in “fungal highway” assays [75,79], and *Stenotrophomonas maltophilia* has been shown to degrade PAHs (e.g., pyrene, benzo(a)pyrene, dibenz(a,h)anthracene) [80,81], while comparative genomics of environmental isolates reveals enriched xenobiotic-degradation equipment [82]. Similarly, *Pseudomonas* spp. possess well-characterized catabolic pathways for PAHs and demonstrate hyphal-mediated transport in soil, thus gaining access to otherwise inaccessible phenanthrene micro-niches and enabling biodegradation [83]; moreover, co-metabolism with naphthalene significantly enhances removal of PAH mixtures in laboratory conditions [84,85]. A plausible role as a hypha-associated bacterium involved in PAH degradation is also hypothesized for the genus *Bosea*, as it is repeatedly recovered and enriched among active degraders under serial phenanthrene selection [56], and members of Hyphomicrobiales (including *Bosea*) are recurrently detected in fungal specimens [86]. The genus *Variovorax* also includes strains capable of consuming phenanthrene, and isolates associated with lichens carry aromatic-degradation genes, consistent with a role in fungal symbiotic contexts [87,88]. Finally, *Chitinophaga* has been demonstrated as endohyphal symbiont in *Fusarium keratinoplasticum*, with measurable effects on fungal substrate use and hyphal density [89].

### 3.5. Strain Characterization

#### 3.5.1. Production of Biosurfactants

Assessing biosurfactant production is essential, as these amphipathic molecules can increase the bioavailability of pollutants and improve the degradative capacity of microorganisms. To this end, biosurfactant production was assessed for 45 strains belonging to 13 species (Table 1). The evaluation was based on the drop collapsing assay, the oil dispersion test, and the emulsification index. For safety reasons, isolates identified as opportunistic human pathogens (i.e., *Aspergillus fumigatus*, *Scedosporium apiospermum*, *Scedosporium dehoogi*, and *E. attenuata*) were excluded from the screening, as they are unsuitable for environmental applications such as bioremediation.

The scoring criteria were defined as follows: The maximum score (+++) was assigned when the supernatant response was comparable to the undiluted Tween-80 control. A response comparable to the 1% diluted Tween-80 control earned a score of ++. A single + was assigned when a minimal but clearly distinguishable response was observed. Finally, the sign − was assigned if the supernatant response was indistinguishable from the negative control (H_2_O_d_). Of the 45 strains screened, 15 exhibited a positive outcome (scoring + or higher) across all three tests, while 11 strains provided a positive response in two out of the three tests (Table 1). Among these, *P. chrysogenum* MUT 7430 warrants particular attention due to its notable performance. It matched undiluted Tween-80 in the DCA and performed comparably to 1% (v/v) Tween-80 in both the ODT and EI. These results align with previous findings identifying *P. chrysogenum* as a versatile producer of both lipopeptides from low-cost substrates and stable surface-active proteins (SAP-Pc) [90,91,92], hence highlighting the species’ potential for cost-effective biotechnological applications. Remarkable activity was also observed in multiple strains of the frequently retrieved *G. pseudocandidus*, whose ability to produce biosurfactants was demonstrated by Eldin and collaborators [71]. Interestingly, all these strains, except MUT 7475, were associated with bacteria: MUT 7477, MUT 7479 and MUT 7482 with putative consortia; MUT 7464, MUT 7480, MUT 7481 and MUT 7505 with *Stenotrophomonas* spp.; MUT 7470 and MUT 7472 with *B. robiniae*; and MUT 7471 with *C. ginsengisegetis*. Notably, strains of *B. robiniae* isolated from hydrocarbon-impacted environments exhibited significant biosurfactant activity [93], similarly to *Stenotrophomonas* spp. [94,95,96] and *Chitinophaga* spp. [97,98]. Finally, positive results were observed in *S. aciculosa* MUT 7449 and *Paracremonium* sp. MUT 7451, although, to our knowledge, there are no reports explicitly documenting biosurfactant production in these genera.

#### 3.5.2. Evaluation of Fungal Growth Under PAH-Selective Conditions

The same strains were screened in 96-well microplates to assess their ability to grow under PAH-selective conditions, where individual PAHs or their mixture were supplied as the only added carbon source. Growth was monitored over three weeks (504 h). This assay should be considered a preliminary screening of fungal growth under PAH-selective conditions rather than direct evidence of pollutant degradation. Indeed, growth in PAH-amended MM may reflect PAH tolerance, putative PAH utilization, or limited use of residual carbon associated with the inoculum. However, the use of inoculum plugs obtained from MM agar plates, together with acetone controls for each strain, reduced this potential bias. Therefore, strains showing significant growth compared with the corresponding acetone control were interpreted as able to grow under PAH-selective conditions, although direct chemical analyses are required to confirm actual PAH depletion and metabolite formation. The growth observed under PAH-selective conditions may involve complementary extracellular and intracellular fungal pathways. Extracellular oxidoreductases, including laccases and lignin-modifying peroxidases, can initiate the oxidation of recalcitrant aromatic structures, while intracellular systems such as cytochrome P450 monooxygenases may further transform oxidized intermediates [28]. However, these mechanisms were not directly assessed here, and fungal degradation efficiency may be influenced by enzyme induction, substrate specificity, pollutant bioavailability, environmental conditions and toxicity [28]. Therefore, the present screening identifies promising strains and fungal–bacterial associations, but PAH depletion assays, metabolite profiling, enzyme activity measurements and gene-expression analyses are required to confirm the pathways involved.

According to the statistical analysis, twelve strains exhibited significantly higher growth than the control in at least one condition and time point (*p* < 0.05; two-way ANOVA, Dunnett’s post hoc test). These included *F. solani* MUT 7447 and MUT 7459, *G. pseudocandidus* MUT 7467, MUT 7469, MUT 7471, MUT 7473, MUT 7475, MUT 7477 and MUT 7484, *P. crustosum* MUT 7445, and *T. versicolor* MUT 7454. In detail, *F. solani* MUT 7447 showed strong growth on PHE at multiple time points (24–504 h) and on BaP and BghiP at selected intervals, whereas several *G. pseudocandidus* strains (MUT 7467, MUT 7469, MUT 7471, MUT 7475, MUT 7477, and MUT 7484) displayed significant growth on PHE, FLUO, BaP, BghiP, and MIX, particularly at later time points (336–504 h). Notably, MUT 7469, MUT 7471, and MUT 7477 were associated with bacteria (*Pseudomonas* sp., *Chitinophaga ginsengisegetis*, and a putative consortium, respectively; Table 2), which can facilitate PAH bioavailability and complement fungal oxidation [65]; this ecological context is consistent with our observation of late-phase growth on HMW PAHs. The significant growth of *F. solani* on PHE and, at selected intervals, on BaP/BghiP reflects its known enzymatic repertoire; ligninolytic enzymes combined with cytochrome P450 catalysis enable transformation of both LMW and HMW PAHs, with experimental degradation of mixed PAHs (including BaP ~71% in 10 days) [99,100,101]. For yeast-like taxa, the significant late-phase growth of *G. pseudocandidus* on PHE/FLUO and, in part, BaP/BghiP/MIX fits the broader framework of P450–epoxide hydrolase–GST pathways documented for PAH transformation in yeasts [102]. Finally, *T. versicolor* MUT 7454 exhibited significant growth on PHE and MIX from 240 h onward. These late but strong responses are consistent with white-rot physiology; for instance, expression of the laccase isomer *TvLac3* gene was upregulated 11 days after treatment with BaP [103]. In general, late growth (≥240–504 h) likely reflects extended adaptation and/or delayed metabolism of the pollutant as a carbon source or an initial lag due to toxicity. Conversely, strains such as *Cladosporium allicinum* MUT 7452 did not show significant growth compared to the control under any tested condition. These findings highlight a subset of fungi, especially strains belonging to *F. solani* and *G. pseudocandidus*, capable of growing under PAH-selective conditions, supporting their potential as promising candidates for subsequent degradation assays and bioremediation-oriented studies. It should be emphasized that the enzymatic basis of this growth response was not directly investigated in the present study. Therefore, the possible involvement of extracellular ligninolytic enzymes and intracellular cytochrome P450-dependent pathways should be regarded as a literature-supported hypothesis rather than a demonstrated mechanism. Future work combining PAH depletion assays, metabolite profiling, enzyme assays and targeted gene-expression analyses will be required to determine whether these strains actively transform PAHs and to identify the specific enzymatic systems involved.

Next, we assessed whether bacterial association relates to biosurfactant production and fungal growth when PAHs were provided as the sole carbon source. The presence of fungal-associated bacteria correlated positively with biosurfactant production (Spearman’s ρ = 0.387, *p* = 0.009, n = 45). On the contrary, no significant correlation was observed between bacterial association and fungal growth, nor between fungal growth and biosurfactant production (*p* > 0.05 for both comparisons; Table S4). The positive correlation between bacterial presence and biosurfactant production is consistent with the known abilities of several genera detected in association with our fungi to produce such compounds [71,94,95,96,97,98] under non-toxic conditions (i.e., in the absence of inhibitory pollutants). In contrast, in microplate assays, bacteria may exert little or no effect on growth. Indeed, pollutant-associated toxicity can suppress bacterial proliferation and activity, limiting biosurfactant synthesis; moreover, bacterial PAH catabolism typically requires cellular uptake, which is also sensitive to toxic stress [104,105]. Together, this framework explains the lack of a positive correlation between biosurfactant production and fungal growth in PAH microplates; when bacterial growth is compromised, biosurfactant synthesis decreases. In addition, the production of biosurfactants was evaluated under stimulatory conditions and did not directly predict fungal growth under PAH-selective conditions. Rather, it should be considered an additional functional trait that may become relevant in soil systems, where pollutant bioavailability, fungal metabolism, and bacterial partners can interact over longer time scales.

## 4. Conclusions

Overall, the present study provides a comprehensive description of the microbial dynamics during the enrichment procedure starting from PAH-contaminated urban soil. By combining culturomics with metabarcoding, we successfully identified a specialized fungal community able to persist and grow under selective conditions imposed by both low- and high-molecular-weight PAHs. A key finding of this work is the high frequency of stable, cross-kingdom associations between fungi and bacteria, underscoring the importance of this interplay in contaminated environments. Our analyses reveal that while associated bacteria correlate with increased biosurfactant production in nutrient-rich conditions, the extreme toxicity of PAHs likely acts as a bottleneck for bacterial growth during active degradation. This suggests that while fungi may act as the primary degraders and “highways” for bacterial dispersal, the synergistic benefits of the consortium may be modulated by the specific bioavailability and toxicity of the pollutants. As demonstrated with metabarcoding approaches, a progressive reduction in microbial diversity and a significant community shift toward specialized taxa occurred as the enrichment advanced, confirming that the procedure effectively filtered the autochthonous population, selecting for the most robust strains. In conclusion, some of the isolated strains exhibit significant potential for bioaugmentation and mycoremediation strategies. Future research should focus on optimizing these fungal–bacterial consortia in soil microcosms and field-like conditions. Further work should characterize their degradative performance, including PAH depletion, metabolite formation and the identification of key enzymatic systems such as laccases, peroxidases and cytochrome P450 monooxygenases, to support the development of effective restoration strategies for polluted ecosystems.

## Figures and Tables

**Table 1:** Results of biosurfactant production assays for each fungal species retrieved in this study. Associated bacteria are indicated where present. DCA = drop collapsing assay; ODT = oil displacement test; EI = emulsification index.

Fungal Species	MUT Number	Associated Bacteria	DCA	ODT	EI
*Cladosporium allicinum*	**7452**		+	+	+
*Cladosporium cladosporioides*	**7444**		-	+	-
*Cladosporium langeronii*	**7441**		-	-	-
*Cladosporium westerdijkia*	**7439**		-	+	+
*Fusarium falciforme*	**7448**		+	-	+
*Fusarium oxysporum*	**7456**		-	-	+
*Fusarium oxysporum*	**7458**		-	++	-
*Fusarium oxysporum*	**7442**		-	-	-
*Fusarium oxysporum*	**7440**		-	-	-
*Fusarium oxysporum*	**7457**		-	-	+
*Fusarium solani*	**7447**		-	-	+
*Fusarium solani*	**7459**		-	-	+
*Galactomyces pseudocandidus*	**7461**		-	-	+
*Galactomyces pseudocandidus*	**7463**	*Bosea robiniae*	+	+	+
*Galactomyces pseudocandidus*	**7462**		-	++	+
*Galactomyces pseudocandidus*	**7464**	*Stenotrophomonas maltophilia*	+	+	++
*Galactomyces pseudocandidus*	**7466**		-	+	+
*Galactomyces pseudocandidus*	**7467**		-	-	-
*Galactomyces pseudocandidus*	**7469**	*Pseudomonas* sp.	-	+	-
*Galactomyces pseudocandidus*	**7470**	*Bosea robiniae*	-	+++	+
*Galactomyces pseudocandidus*	**7471**	*Chitinophaga ginsengisegetis*	+	++	+
*Galactomyces pseudocandidus*	**7472**	*Bosea robiniae*	+	++	++
*Galactomyces pseudocandidus*	**7474**	*Stenotrophomonas* sp.	+	+++	-
*Galactomyces pseudocandidus*	**7475**		+	+	++
*Galactomyces pseudocandidus*	**7476**	Putative consortium	+	+	+
*Galactomyces pseudocandidus*	**7477**	Putative consortium	++	++	++
*Galactomyces pseudocandidus*	**7478**	*Stenotrophomonas* sp.	-	-	+
*Galactomyces pseudocandidus*	**7479**	Putative consortium	+	+	++
*Galactomyces pseudocandidus*	**7480**	*Stenotrophomonas* sp.	+	+	+
*Galactomyces pseudocandidus*	**7481**	*Stenotrophomonas* sp.	-	-	+
*Galactomyces pseudocandidus*	**7482**	Putative consortium	+	++	+
*Galactomyces pseudocandidus*	**7483**	Putative consortium	-	-	+
*Galactomyces pseudocandidus*	**7484**		-	+	+
*Galactomyces pseudocandidus*	**7505**	*Stenotrophomonas* sp.	+	+++	+
*Galactomyces pseudocandidus*	**7485**	*Stenotrophomonas indicatrix*	-	+	++
*Galactomyces pseudocandidus*	**7465**		-	+	+
*Paracremonium* sp.	**7451**		++	+	+
*Paracremonium* sp.	**7450**		++	+	-
*Penicillium chrysogenum*	**7430**		+++	++	++
*Penicillium crustosum*	**7445**		-	-	-
*Stilbella aciculosa*	**7449**		+++	+	-
*Stilbella aciculosa*	**7436**	*Chitinophaga arvensicola*	+	++	+
*Stilbella aciculosa*	**7438**		-	-	+
*Stilbella aciculosa*	**7453**	*Chitinophaga ginsengisegetis*	-	+	+
*Trametes versicolor*	**7454**	*Bosea robiniae*	-	+	-

**Table 2:** Statistical comparison of fungal strain growth over time under PAH-selective conditions. For each strain and PAH condition, significance refers to the comparison between fungal growth in PAH-amended medium and the corresponding acetone control at each time point.

Strain		Hours	24	48	72	168	240	336	504
	PAH								
*Cladosporium allicinum* MUT 7452	PHE		ns	ns	ns	ns	ns	*	*
	FLUO		ns	ns	ns	ns	ns	ns	ns
	BaP		ns	ns	ns	ns	ns	ns	ns
	BghiP		ns	ns	ns	ns	ns	ns	ns
	MIX		ns	ns	ns	ns	ns	ns	ns
*Fusarium solani* MUT 7447	PHE		**	***	***	****	**	****	**
	FLUO		ns	ns	ns	ns	ns	***	ns
	BaP		***	ns	**	ns	ns	****	*
	BghiP		ns	**	ns	ns	ns	***	ns
	MIX		ns	ns	ns	ns	ns	***	ns
*Fusarium solani* MUT 7459	PHE		ns	ns	ns	ns	**	**	*
	FLUO		ns	ns	ns	ns	ns	ns	ns
	BaP		**	ns	**	ns	*	*	ns
	BghiP		*	ns	ns	*	*	*	*
	MIX		*	ns	ns	ns	ns	ns	ns
*Galactomyces pseudocandidus* MUT 7467	PHE		**	**	**	NA	ns	ns	****
	FLUO		**	****	**	NA	ns	ns	ns
	BaP		ns	ns	ns	NA	*	ns	****
	BghiP		*	**	*	NA	ns	ns	ns
	MIX		***	****	ns	NA	ns	ns	ns
*Galactomyces pseudocandidus* MUT 7469 *Pseudomonas* sp.	PHE		**	**	*	*	*	*	ns
	FLUO		ns	ns	ns	ns	ns	ns	ns
	BaP		ns	ns	ns	ns	ns	*	ns
	BghiP		ns	ns	ns	ns	ns	ns	ns
	MIX		ns	ns	ns	ns	*	*	ns
*Galactomyces pseudocandidus* MUT 7471 *Chitinophaga ginsengisegetis*	PHE		ns	ns	*	ns	ns	ns	*
	FLUO		ns	*	ns	ns	ns	ns	ns
	BaP		ns	ns	ns	**	****	**	ns
	BghiP		ns	ns	ns	***	****	****	****
	MIX		ns	ns	ns	ns	**	*	ns
*Galactomyces pseudocandidus* MUT 7473	PHE		*	ns	*	ns	ns	ns	ns
	FLUO		ns	ns	*	ns	ns	ns	ns
	BaP		***	ns	***	ns	ns	ns	ns
	BghiP		**	ns	****	ns	ns	ns	ns
	MIX		ns	ns	*	ns	ns	ns	ns
*Galactomyces pseudocandidus* MUT 7475	PHE		****	***	****	ns	ns	ns	**
	FLUO		ns	ns	ns	*	**	ns	ns
	BaP		ns	ns	*	*	**	*	*
	BghiP		ns	ns	ns	**	***	****	****
	MIX		ns	*	ns	*	**	***	**
*Galactomyces pseudocandidus* MUT 7477 Putative consortium	PHE		ns	ns	ns	NA	ns	*	***
	FLUO		*	ns	ns	NA	ns	ns	ns
	BaP		***	***	*	NA	ns	**	***
	BghiP		ns	ns	ns	NA	**	****	*
	MIX		ns	*	ns	NA	ns	ns	ns
*Galactomyces pseudocandidus* MUT 7484	PHE		ns	**	***	ns	ns	ns	**
	FLUO		ns	***	*	ns	ns	ns	ns
	BaP		ns	**	**	ns	ns	ns	ns
	BghiP		ns	ns	ns	ns	***	***	**
	MIX		ns	ns	ns	ns	ns	ns	ns
*Penicillium crustosum* MUT 7445	PHE		**	ns	ns	ns	ns	ns	ns
	FLUO		ns	**	***	*	ns	ns	ns
	BaP		*	ns	**	*	ns	ns	ns
	BghiP		ns	**	ns	ns	*	ns	*
	MIX		ns	ns	ns	ns	ns	ns	ns
*Trametes versicolor* MUT 7454	PHE		ns	ns	ns	ns	*	****	****
	FLUO		ns	ns	ns	ns	ns	ns	ns
	BaP		ns	ns	ns	ns	ns	ns	ns
	BghiP		ns	ns	ns	ns	****	****	****
	MIX		ns	ns	ns	ns	**	****	****

* *p* < 0.05, ** *p* < 0.01, *** *p* < 0.001, **** *p* < 0.0001; red asterisks indicate significantly lower growth than the control. NA = not available, ns = not significant.

## Data Availability

The data generated in the study are openly available in NCBI GenBank at https://www.ncbi.nlm.nih.gov/genbank (accessed on 20 June 2026) and Sequence Read Archive (SRA) at https://www.ncbi.nlm.nih.gov/sra (accessed on 20 June 2026).

## Supplementary Materials

The following supporting information can be downloaded at: https://www.mdpi.com/article/10.3390/jof12070469/s1, Figure S1: Rarefaction curve of fungal and bacterial sample sequencing. Figure S2: Chao1 and Shannon alpha diversity of fungal and bacterial populations. Figure S3: Barplots showing the relative abundance of the most represented fungal phyla, classes and orders. Figure S4: Concentration of PHE in percentages at the beginning (T0) and at the end (T7) of each enrichment step. Table S1: Alpha diversity measures of the sequenced samples. Table S2: Taxonomy of the retrieved fungal isolates. Table S3: Fungal taxa retrieved from different conditions. Table S4: Correlation between biosurfactant production, bacterial presence and fungal growth.

## Abbreviations

The following abbreviations are used in this manuscript:

BaP	Benzo(a)pyrene
BGHI	Benzo(g,h,i)perylene
CTRL	Control
FLUO	Fluoranthene
MEA	Malt extract agar
MIX	Mixture of the four PAHs
MM	Mineral medium
PAH	Polycyclic aromatic hydrocarbon
PHE	Phenanthrene

## References

[1] Saccá M., Caracciolo A., Di Lenola M., Grenni P. (2017). Ecosystem services provided by soil microorganisms. Proceedings of the Soil Biological Communities and Ecosystem Resilience.

[2] Sokol N., Slessarev E., Marschmann G., Nicolas A., Blazewicz S., Brodie E., Firestone M., Foley M., Hestrin R., Hungate B. (2022). Life and death in the soil microbiome: How ecological processes influence biogeochemistry. Nat. Rev. Microbiol..

[3] Anikwe M., Ife K. (2023). The role of soil ecosystem services in the circular bioeconomy. Front. Soil Sci..

[4] Cachada A., da Silva E., Duarte A., Pereira R. (2016). Risk assessment of urban soils contamination: The particular case of polycyclic aromatic hydrocarbons. Sci. Total Environ..

[5] Wang C., Wu S., Zhou S., Sill Y., Song J. (2017). Characteristics and source identification of polycyclic aromatic hydrocarbons (PAHs) in urban soils: A Review. Pedosphere.

[6] Heywood E., Wright J., Wienburg C., Black H., Long S., Osborn D., Spurgeon D. (2006). Factors influencing the national distribution of polycyclic aromatic hydrocarbons and polychlorinated biphenyls in British soils. Environ. Sci. Technol..

[7] Conte P., Zena A., Pilidis G., Piccolo A. (2001). Increased retention of polycyclic aromatic hydrocarbons in soils induced by soil treatment with humic substances. Environ. Pollut..

[8] Sakshi, Singh S., Haritash A. (2019). Polycyclic aromatic hydrocarbons: Soil pollution and remediation. Int. J. Environ. Sci. Technol..

[9] Weissenfels W., Klewer H., Langhoff J. (1992). Adsorption of polycyclic aromatic-hydrocarbons (PAHS) by soil particles: Influence on biodegradability and biotoxicity. Appl. Microbiol. Biotechnol..

[10] Haritash A., Kaushik C. (2009). Biodegradation aspects of polycyclic aromatic hydrocarbons (PAHs): A review. J. Hazard. Mater..

[11] Imam A., Suman S., Kanaujia P., Ray A. (2022). Biological machinery for polycyclic aromatic hydrocarbons degradation: A review. Bioresour. Technol..

[12] Godoy P., Reina R., Calderón A., Wittich R., García-Romera I., Aranda E. (2016). Exploring the potential of fungi isolated from PAH-polluted soil as a source of xenobiotics-degrading fungi. Environ. Sci. Pollut. Res..

[13] Ijoma G., Tekere M. (2017). Potential microbial applications of co-cultures involving ligninolytic fungi in the bioremediation of recalcitrant xenobiotic compounds. Int. J. Environ. Sci. Technol..

[14] Miglani R., Parveen N., Kumar A., Ansari M., Khanna S., Rawat G., Panda A., Bisht S., Upadhyay J., Ansari M. (2022). Degradation of Xenobiotic Pollutants: An Environmentally Sustainable Approach. Metabolites.

[15] Kuppan N., Padman M., Mahadeva M., Srinivasan S., Devarajan R. (2024). A comprehensive review of sustainable bioremediation techniques: Eco friendly solutions for waste and pollution management. Waste Manag. Bull..

[16] Singh B., Christina E. (2022). Indigenous microorganisms as an effective tool for in situ bioremediation. Relationship Between Microbes and the Environment for Sustainable Ecosystem Services.

[17] Fallahi M., Sarempour M., Gohari A. (2023). Potential biodegradation of polycyclic aromatic hydrocarbons (PAHs) and petroleum hydrocarbons by indigenous fungi recovered from crude oil-contaminated soil in Iran. Sci. Rep..

[18] González-Abradelo D., Pérez-Llano Y., Peidro-Guzmán H., Sánchez-Carbente M., Folch-Mallol J., Aranda E., Vaidyanathan V., Cabana H., Gunde-Cimerman N., Batista-García R. (2019). First demonstration that ascomycetous halophilic fungi (*Aspergillus sydowii* and *Aspergillus destruens*) are useful in xenobiotic mycoremediation under high salinity conditions. Bioresour. Technol..

[19] Pozdnyakova N., Nikiforova S., Turkovskaya O. (2010). Influence of PAHs on ligninolytic enzymes of the *fungus Pleurotus ostreatus* D1. Cent. Eur. J. Biol..

[20] Liu P., Wen S., Zhu S., Hu X., Wang Y. (2025). Microbial degradation of soil organic pollutants: Mechanisms, challenges, and advances in forest ecosystem management. Processes.

[21] Agnello A., Bagard M., van Hullebusch E., Esposito G., Huguenot D. (2016). Comparative bioremediation of heavy metals and petroleum hydrocarbons co-contaminated soil by natural attenuation, phytoremediation, bioaugmentation and bioaugmentation-assisted phytoremediation. Sci. Total Environ..

[22] Muter O. (2023). Current Trends in Bioaugmentation Tools for Bioremediation: A critical review of advances and knowledge gaps. Microorganisms.

[23] Spini G., Spina F., Poli A., Blieux A., Regnier T., Gramellini C., Varese G., Puglisi E. (2018). Molecular and microbiological insights on the enrichment procedures for the isolation of petroleum degrading bacteria and fungi. Front. Microbiol..

[24] Zhu S., Li M., Qian T., Chen J., Pan T. (2025). Influence of surfactants on interfacial microbial degradation of hydrophobic organic compounds. Catalysts.

[25] Aparna A., Srinikethan G., Hegde S. (2011). Effect of addition of biosurfactant produced by *Pseudomonas* sps. on biodegradation of crude oil. Proceedings of the Environmental Science and Technology.

[26] Santos B., Jesus M., Mata F., Prado A., Vieira I., Ramos L., López J., Vaz-Velho M., Ruzene D., Silva D. (2023). Use of Agro-Industrial Waste for Biosurfactant Production: A comparative study of hemicellulosic liquors from corncobs and sunflower stalks. Sustainability.

[27] Ibrahim A., Oginga B., Zhang Y., Ling W., Tang L., Elatafi E., Abady M., Gao Y. (2025). Bioremediation of soils with emerging organic contaminants using immobilized microorganisms. Environ. Technol. Innov..

[28] Khan M. (2025). Microbial remediation of agrochemical-contaminated soils: Enzymatic mechanisms, quorum sensing, and emerging opportunities. Integr. Environ. Assess. Manag..

[29] Giunchino F., Mucciarelli M., Malandrino M., Sordello F., Lanfranco L., Primo L., Calza P. (2026). Assessing PTEs in a polluted urban green environment and proposing sustainable approaches for its recovery. Ecol. Eng..

[30] Poli A., Bongiovanni D., Stefanini I., Crespi M., Giunchino F., Morel E., Calza P., Varese G., Prigione V. (2026). Autochthonous microorganisms of a soil contaminated by polycyclic aromatic hydrocarbons: Allies or silent threats?. Biodivers. Conserv..

[31] White T.J., Bruns T., Lee S., Taylor J. (1990). Amplification and direct sequencing of fungal ribosomal RNA genes for phylogenetics. PCR Protoc. A Guide Methods Appl..

[32] Ihrmark K., Bödeker I., Cruz-Martinez K., Friberg H., Kubartova A., Schenck J., Strid Y., Stenlid J., Brandström-Durling M., Clemmensen K. (2012). New primers to amplify the fungal ITS2 region—Evaluation by 454-sequencing of artificial and natural communities. FEMS Microbiol. Ecol..

[33] Klindworth A., Pruesse E., Schweer T., Peplies J., Quast C., Horn M., Glöckner F. (2013). Evaluation of general 16S ribosomal RNA gene PCR primers for classical and next-generation sequencing-based diversity studies. Nucleic Acids Res..

[34] Bolyen E., Rideout J., Dillon M., Bokulich N., Abnet C., Al-Ghalith G., Alexander H., Alm E., Arumugam M., Asnicar F. (2019). Reproducible, interactive, scalable and extensible microbiome data science using QIIME 2. Nat. Biotechnol..

[35] Callahan B., McMurdie P., Rosen M., Han A., Johnson A., Holmes S. (2016). DADA2: High-resolution sample inference from Illumina amplicon data. Nat. Methods.

[36] Quast C., Pruesse E., Yilmaz P., Gerken J., Schweer T., Yarza P., Peplies J., Glöckner F. (2013). The SILVA ribosomal RNA gene database project: Improved data processing and web-based tools. Nucleic Acids Res..

[37] Vilgalys R., Hester M. (1990). Rapid genetic identification and mapping of enzymatically amplified ribosomal DNA from several Cryptococcus species. J. Bacteriol..

[38] Glass N.L., Donaldson G.C. (1995). Development of primer sets designed for use with the PCR to amplify conserved genes from filamentous ascomycetes. Appl. Environ. Microbiol..

[39] Carbone I., Kohn L.M. (1999). A method for designing primer sets for speciation studies in filamentous ascomycetes. Mycologia.

[40] O’Donnell K. (2000). Molecular phylogeny of the *Nectria haematococca-Fusarium solani* species complex. Mycologia.

[41] Lane D.J., Stackebrandt E., Goodfellow M. (1991). 16S/23S rRNA Sequencing. Nucleic Acid Techniques in Bacterial Systematic.

[42] Poli A., Zanellati A., Piano E., Biagioli F., Coleine C., Nicolosi G., Selbmann L., Isaia M., Prigione V., Varese G. (2024). Cultivable fungal diversity in two karstic caves in Italy: Under-investigated habitats as source of putative novel taxa. Sci. Rep..

[43] Poli A., Lazzari A., Prigione V., Voyron S., Spadaro D., Varese G.C. (2016). Influence of plant genotype on the cultivable fungi associated to tomato rhizosphere and roots in different soils. Fungal Biol..

[44] Fidalgo-Jiménez A., Danie H., Evrard P., Decock C., Lachance M. (2008). *Metschnikowia cubensis* sp. nov., a yeast species isolated from flowers in Cuba. Int. J. Syst. Evol. Microbiol..

[45] Bertout S., Drakulovski P., Kouanfack C., Krasteva D., Ngouana T., Dunyach-Rémy C., Dongtsa J., Aghokeng A., Delaporte E., Koulla-Shiro S. (2013). Genotyping and antifungal susceptibility testing of *Cryptococcus neoformans* isolates from Cameroonian HIV-positive adult patients. Clin. Microbiol. Infect..

[46] Bodour A., Miller-Maier R. (1998). Application of a modified drop-collapse technique for surfactant quantitation and screening of biosurfactant-producing microorganisms. J. Microbiol. Methods.

[47] Youssef N., Duncan K., Nagle D., Savage K., Knapp R., McInerney M. (2004). Comparison of methods to detect biosurfactant production by diverse microorganisms. J. Microbiol. Methods.

[48] Morikawa M., Hirata Y., Imanaka T. (2000). A study on the structure-function relationship of lipopeptide biosurfactants. Biochim. Biophys. Acta-Mol. Cell Biol. Lipids.

[49] Walter V., Syldatk C., Hausmann R. (2010). Screening concepts for the isolation of biosurfactant producing microorganisms. Biosurfactants.

[50] Al-hazmi M., Moussa T., Alhazmi N. (2023). Statistical optimization of biosurfactant production from *Aspergillus niger* SA1 fermentation process and mathematical modeling. J. Microbiol. Biotechnol..

[51] McNab E., Rether A., Hsiang T. (2023). Development of a microplate absorbance assay for assessing fungicide sensitivity of filamentous fungi and comparison to an amended agar assay. J. Microbiol. Methods.

[52] Wang C., Wu H., Zhao W., Zhu B., Yang J. (2024). Effects of Polycyclic aromatic hydrocarbons on soil bacterial and fungal communities in soils. Diversity.

[53] Gréau L., Blaudez D., Le Cordier H., Fornasier F., Cébron A. (2023). Taxonomic and functional responses of soil and root bacterial communities associated with poplar exposed to a contamination gradient of phenanthrene. FEMS Microbiol. Ecol..

[54] Egidi E., Delgado-Baquerizo M., Plett J., Wang J., Eldridge D., Bardgett R., Maestre F., Singh B. (2019). A few Ascomycota taxa dominate soil fungal communities worldwide. Nat. Commun..

[55] Zhang S., Wang Q., Wan R., Xie S. (2011). Changes in bacterial community of anthracene bioremediation in municipal solid waste composting soil. J. Zhejiang Univ. -Sci. B.

[56] Zhou G., Qiao H., Liu Y., Yu X., Niu X. (2024). High phenanthrene degrading efficiency by different microbial compositions construction. Front. Microbiol..

[57] Shen Q., Fu W., Chen B., Zhang X., Xing S., Ji C., Zhang X. (2023). Community response of soil microorganisms to combined contamination of polycyclic aromatic hydrocarbons and potentially toxic elements in a typical coking plant. Front. Microbiol..

[58] Luo C., Guan G., Dai Y., Cai X., Huang Q., Li J., Zhang G. (2024). Determination of soil phenanthrene degradation through a fungal-bacterial consortium. Appl. Environ. Microbiol..

[59] Saito A., Iwabuchi T., Harayama S. (2000). A novel phenanthrene dioxygenase from Nocardioides sp. strain KP7: Expression in Escherichia coli. J. Bacteriol..

[60] Nzila A., Musa M.M., Afuecheta E., Al-Thukair A., Sankaran S., Xiang L., Li Q.X. (2023). Benzo [a] pyrene biodegradation by multiple and individual mesophilic bacteria under axenic conditions and in soil samples. Int. J. Environ. Res. Public Health.

[61] Pozdnyakova N., Muratova A., Bondarenkova A., Turkovskaya O. (2023). Degradation of a Model mixture of PAHs by bacterial–fungal co-cultures. Front. Biosci. -Elite.

[62] Aranda E., Godoy P., Reina R., Badia-Fabregat M., Rosell M., Marco-Urrea E., García-Romera I. (2017). Isolation of of Ascomycota fungi with capability to transform PAHs: Insights into the biodegradation mechanisms of *Penicillium oxalicum*. Int. Biodeterior. Biodegrad..

[63] Gao J., Qi M., Wang X., Feng X., Li J., Zhang G., Feng S., Yang Z., Ning G. (2025). Combined induction by Cu(II) and veratrole enhances the degradation of high molecular weight polyaromatic hydrocarbons by *Fusarium dlaminii* ZH-H2. Ecotoxicol. Environ. Saf..

[64] Wang X., Gong Z., Li P., Zhang L., Hu X. (2008). Degradation of pyrene and benzo(a) pyrene in contaminated soil by immobilized fungi. Environ. Eng. Sci..

[65] Thion C., Cébron A., Beguiristain T., Leyval C. (2012). PAH biotransformation and sorption by *Fusarium solani* and *Arthrobacter oxydans* isolated from a polluted soil in axenic cultures and mixed co-cultures. Int. Biodeterior. Biodegrad..

[66] Potin O., Veignie E., Rafin C. (2004). Biodegradation of polycyclic aromatic hydrocarbons (PAHs) by *Cladosporium sphaerospermum* isolated from an aged PAH contaminated soil. FEMS Microbiol. Ecol..

[67] Birolli W., Santos D., Alvarenga N., Garcia A., Romao L., Porto A. (2018). Biodegradation of anthracene and several PAHs by the marine-derived fungus *Cladosporium* sp. CBMAI 1237. Mar. Pollut. Bull..

[68] Egbewale S., Kumar A., Olasehinde T., Mokoena M., Olaniran A. (2024). Anthracene detoxification by Laccases from indigenous fungal strains *Trichoderma lixii* FLU1 and *Talaromyces pinophilus* FLU12. Biodegradation.

[69] Potin O., Rafin C., Veignie E. (2004). Bioremediation of an aged polycyclic aromatic hydrocarbons (PAHs)-contaminated soil by filamentous fungi isolated from the soil. Int. Biodeterior. Biodegrad..

[70] Ghosal D., Ghosh S., Dutta T., Ahn Y. (2016). Current state of knowledge in microbial degradation of polycyclic aromatic hydrocarbons (PAHs): A Review. Front. Microbiol..

[71] Eldin A., Kamel Z., Hossam N. (2019). Isolation and genetic identification of yeast producing biosurfactants, evaluated by different screening methods. Microchem. J..

[72] Kamilari E., Stanton C., Reen F., Ross R. (2023). Uncovering the biotechnological importance of *Geotrichum candidum*. Foods.

[73] Obi L., Atagana H., Adeleke R., Maila M., Bamuza-Pemu E. (2020). Potential microbial drivers of biodegradation of polycyclic aromatic hydrocarbons in crude oil sludge using a composting technique. J. Chem. Technol. Biotechnol..

[74] Kohlmeier S., Smits T., Ford R., Keel C., Harms H., Wick L. (2005). Taking the fungal highway: Mobilization of pollutant-degrading bacteria by fungi. Environ. Sci. Technol..

[75] Simon A., Bindschedler S., Job D., Wick L., Filippidou S., Kooli W., Verrecchia E., Junier P. (2015). Exploiting the fungal highway: Development of a novel tool for the in situ isolation of bacteria migrating along fungal mycelium. FEMS Microbiol. Ecol..

[76] Richter I., Buettner H., Hertweck C. (2025). Endofungal bacteria as hidden facilitators of biotic interactions. ISME J..

[77] Worrich A., König S., Miltner A., Banitz T., Centler F., Frank K., Thullner M., Harms H., Kästner M., Wick L. (2016). Mycelium-like networks increase bacterial dispersal, growth, and biodegradation in a model ecosystem at various water potentials. Appl. Environ. Microbiol..

[78] Ksiazek-Trela P., Figura D., Wezka D., Szpyrka E. (2024). Degradation of a mixture of 13 polycyclic aromatic hydrocarbons by commercial effective microorganisms. Open Life Sci..

[79] Junier P., Cailleau G., Palmieri I., Vallotton C., Trautschold O., Junier T., Paul C., Bregnard D., Palmieri F., Estoppey A. (2021). Democratization of fungal highway columns as a tool to investigate bacteria associated with soil fungi. FEMS Microbiol. Ecol..

[80] Juhasz A., Stanley G., Britz M. (2000). Microbial degradation and detoxification of high molecular weight polycyclic aromatic hydrocarbons by Stenotrophomonas maltophilia strain VUN 10,003. Lett. Appl. Microbiol..

[81] Singh A., Kumar K., Pandey A., Sharma A., Singh S., Kumar K., Arora A., Nain L. (2015). pyrene degradation by biosurfactant producing bacterium *Stenotrophomonas maltophilia*. Agric. Res..

[82] Xiao Y., Jiang R., Wu X., Zhong Q., Li Y., Wang H. (2021). Comparative genomic analysis of *Stenotrophomonas maltophilia* strain W18 reveals its adaptative genomic features for degrading polycyclic aromatic hydrocarbons. Microbiol. Spectr..

[83] Wick L., Remer R., Würz B., Reichenbach J., Braun S., Schärfer F., Harms H. (2007). Effect of fungal hyphae on the access of bacteria to phenanthrene in soil. Environ. Sci. Technol..

[84] Cao H., Zhang X., Wang S., Liu J., Han D., Zhao B., Wang H. (2021). Insights into mechanism of the naphthalene-enhanced biodegradation of phenanthrene by *Pseudomonas* sp. SL-6 based on omics analysis. Front. Microbiol..

[85] Li B., Liu H., Liu X., Han L., Yang J., Kang L., Tang L., Qian T. (2024). Naphthalene enhances polycyclic aromatic hydrocarbon biodegradation by *Pseudomonas aeruginosa* in soil and water: Effect and mechanism. Water.

[86] Escudero-Leyva E., Belle M., DadkhahTehrani A., Culver J., Araya-Salas M., Kutza J., Goldson N., Chavarría M., Chaverri P. (2025). Genomic insights reveal community structure and phylogenetic associations of endohyphal bacteria and viruses in fungal endophytes. Environ. Microbiome.

[87] Liu J., Cui Z., Hao T., Li Y., Luan X., Feng K., Zheng L. (2023). Characterization and hydrocarbon degradation potential of *Variovorax* sp. strain N23 isolated from the antarctic soil. Microbiol. Res..

[88] Ghimire N., Kim B., Lee C., Oh T. (2022). Comparative genome analysis among *Variovorax* species and genome guided aromatic compound degradation analysis emphasizing 4-hydroxybenzoate degradation in *Variovorax* sp. PAMC26660. BMC Genom..

[89] Shaffer J., U’Ren J., Gallery R., Baltrus D., Arnold A. (2017). An endohyphal bacterium (Chitinophaga, Bacteroidetes) alters carbon source use by *Fusarium keratoplasticum* (*F. solani* Species Complex, Nectriaceae). Front. Microbiol..

[90] Cicatiello P., Stanzione I., Dardano P., De Stefano L., Birolo L., De Chiaro A., Monti D., Petruk G., D’Errico G., Giardina P. (2019). Characterization of a surface-active protein extracted from a marine strain of *Penicillium chrysogenum*. Int. J. Mol. Sci..

[91] Gautam G., Mishra V., Verma P., Pandey A.K., Negi S. (2014). A cost effective strategy for production of bio-surfactant from locally isolated Penicillium chrysogenum SNP5 and its applications. J. Bioprocess. Biotech..

[92] Khan A., Tanveer S., Kiyani A., Barros R., Iqbal M., Yousaf S. (2023). Biosurfactant-producing *Aspergillus*, *Penicillium*, and *Candida* Performed higher biodegradation of diesel oil than a non-producing fungal strain. Appl. Biochem. Microbiol..

[93] Ruggeri C., Franzetti A., Bestetti G., Caredda P., La Colla P., Pintus M., Sergi S., Tamburini E. (2009). Isolation and characterisation of surface active compound-producing bacteria from hydrocarbon-contaminated environments. Int. Biodeterior. Biodegrad..

[94] Gargouri B., Contreras M., Ammar S., Segura-Carretero A., Bouaziz M. (2017). Biosurfactant production by the crude oil degrading *Stenotrophomonas* sp. B-2: Chemical characterization, biological activities and environmental applications. Environ. Sci. Pollut. Res..

[95] Patel K., Patel M. (2020). Improving bioremediation process of petroleum wastewater using biosurfactants producing *Stenotrophomonas* sp. S1VKR-26 and assessment of phytotoxicity. Bioresour. Technol..

[96] Larik I., Qazi M., Phulpoto A., Haleem A., Ahmed S., Kanhar N. (2019). Stenotrophomonas maltophilia strain 5DMD: An efficient biosurfactant-producing bacterium for biodegradation of diesel oil and used engine oil. Int. J. Environ. Sci. Technol..

[97] Yuan C., Wang J., Wu J., Li Q. (2025). Degradation of puffed feather to produce functional biosurfactants by *Chitinophaga eiseniae* 4 H. Process Biochem..

[98] Udume O.A., Stanley H.O., Abu G.O. (2021). Hydrocarbon solubilization by oil and cellulose-degrading *Chitinophaga terrae* isolated from the rumen. GSC Biol. Pharm. Sci..

[99] Hammel K. (1995). Mechanisms for polycyclic aromatic hydrocarbon degradation by ligninolytic fungi. Environ. Health Perspect..

[100] Aydin S., Karaçay H., Shahi A., Gökçe S., Ince B., Ince O. (2017). Aerobic and anaerobic fungal metabolism and omics insights for increasing polycyclic aromatic hydrocarbons biodegradation. Fungal Biol. Rev..

[101] Dai Y., Liu R., Chen J., Li N. (2022). Bioremediation of HMW-PAHs-contaminated soils by rhizosphere microbial community of Fire Phoenix plants. Chem. Eng. J..

[102] Padilla-Garfias F., Araiza-Villanueva M., Calahorra M., Sánchez N., Peña A. (2024). Advances in the degradation of polycyclic aromatic hydrocarbons by yeasts: A Review. Microorganisms.

[103] Sun Y., Li Y., Liang H., Li M., Liu Y., Wang L., Lai W., Tang T., Diao Y., Bai Y. (2023). Distinct laccase expression and activity profiles of Trametes versicolor facilitate degradation of benzo[a]pyrene. Front. Bioeng. Biotechnol..

[104] Winding A., Modrzynski J., Christensen J., Brandt K., Mayer P. (2019). Soil bacteria and protists show different sensitivity to polycyclic aromatic hydrocarbons at controlled chemical activity. FEMS Microbiol. Lett..

[105] Das N., Chandran P. (2011). Microbial degradation of petroleum hydrocarbon contaminants: An overview. Biotechnol. Res. Int..

