# Supplementary material for: When Fungi Meet Bacteria: Cross-Kingdom Assembly and Bioremediation Potential Under PAH Stress
Source: J Fungi (Basel). 2026 Jun 25;12(7):469. doi: 10.3390/jof12070469 (PMC13413076; doi:10.3390/jof12070469)
Supplement: Supplementary file 1 [file jof-12-00469-s001.zip › jof-4371055-supplementary.pdf]

When Fungi Meet Bacteria: Cross-Kingdom Assembly and Bioremediation Potential Under PAH Stress

Anna Poli [1,2], Andrea L. Marchitelli [1], Irene Stefanini [1,2,*], Marina Bambi [1], Francesco Giunchino [2,3], Paola Calza [2,3], Giovanna Cristina Varese [1,2] and Valeria Prigione [1,2,*]

[1] Department of Life Sciences and Systems Biology, University of Torino, Viale Mattioli 25, 10125 Torino, Italy; anna.poli@unito.it (A.P.)
[2] National Biodiversity Future Center—NBFC, Piazza Marina 61, 90133 Palermo, Italy
[3] Department of Chemistry, University of Torino, Via Pietro Giuria 7, 10125 Torino, Italy
* Correspondence: irene.stefanini@unito.it (I.S.); valeria.prigione@unito.it (V.P.)

This document includes:

Figure S1: Rarefaction curve of **A)** fungal and **B)** bacterial samples sequencing.

Figure S2: Chao1 and Shannon alpha diversity of fungal and bacterial populations. **A)** Chao1 alpha diversity in fungal populations in different enrichment treatments at different steps of the process. **B)** Chao1 alpha diversity in bacterial populations in different enrichment treatments at different steps of the process. **C)** Shannon alpha diversity in fungal populations in different enrichment treatments at different steps of the process. **D)** Shannon alpha diversity in bacterial populations in different enrichment treatments at different steps of the process. Differences among groups of samples alpha diversities (Wilcoxon-Mann-Whitney test p value<0.05) are shown with different letters in the plot.

Figure S3: Barplots showing the relative abundance of the most represented fungal phyla, classes and orders.

Figure S4: Concentration of PHE in percentage at the beginning (T0) and at the end (T7) of each enrichment step. Different lowercases indicate statistical significance (unpaired t-test; p<0.05)

Table S1: alpha diversity measures of the sequenced samples. PHE=phenanthrene; Fluo=fluoranthene; BaP=benzo(a)pyrene; BGHI=benzo(g,h,i)perilyene; MIX=Mix of the four PAHs. OBS.= Observed diversity.

Table S2: Taxonomy of the retrieved fungal isolates.

Table S3: Fungal taxa retrieved from different conditions. For each taxa associated bacteria are reported. Equal upper case letters within the same taxon indicate more isolates of the same strain.

Table S4: Correlation between Biosurfactant production, bacterial presence and fungal growth.

Figure S1. Rarefaction curve of **A)** fungal and **B)** bacterial samples sequencing.

A

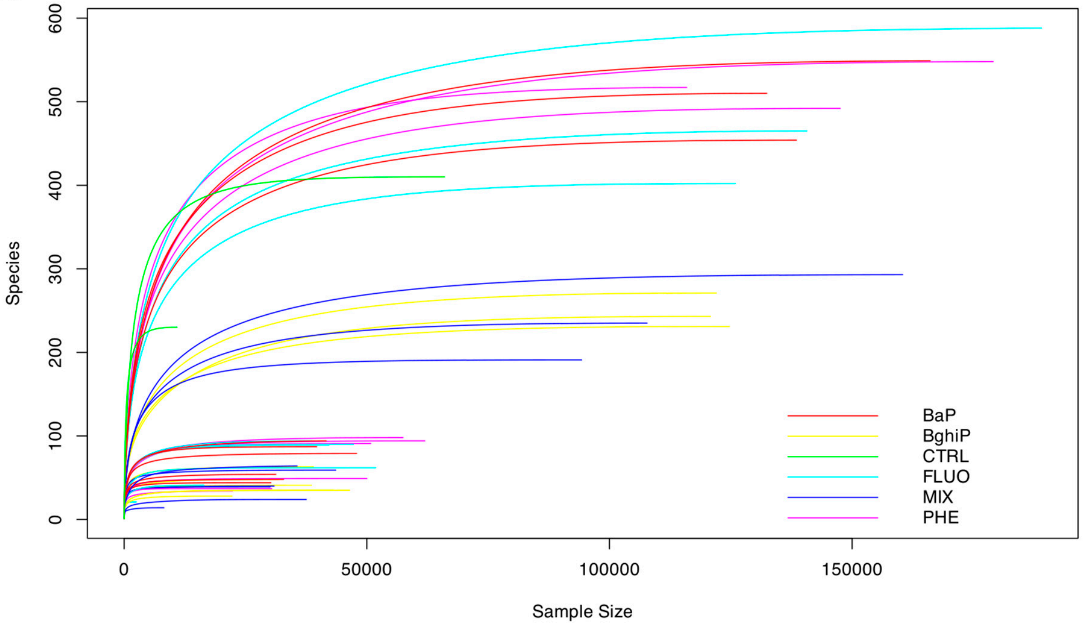

B

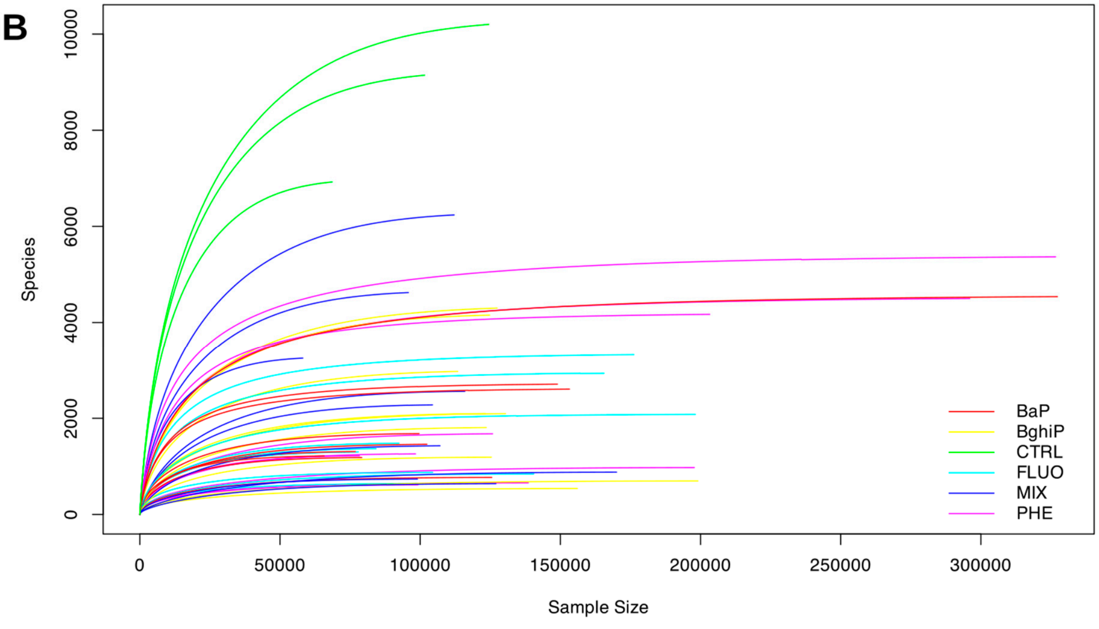

Figure S2. Chao1 and Shannon alpha diversity of fungal and bacterial populations. **A)** Chao1 alpha diversity in fungal populations in different enrichment treatments at different steps of the process. **B)** Chao1 alpha diversity in bacterial populations in different enrichment treatments at different steps of the process. **C)** Shannon alpha diversity in fungal populations in different enrichment treatments at different steps of the process. **D)** Shannon alpha diversity in bacterial populations in different enrichment treatments at different steps of the process. Differences among groups of samples alpha diversities (Wilcoxon-Mann-Whitney test p value<0.05) are shown with different letters in the plot.

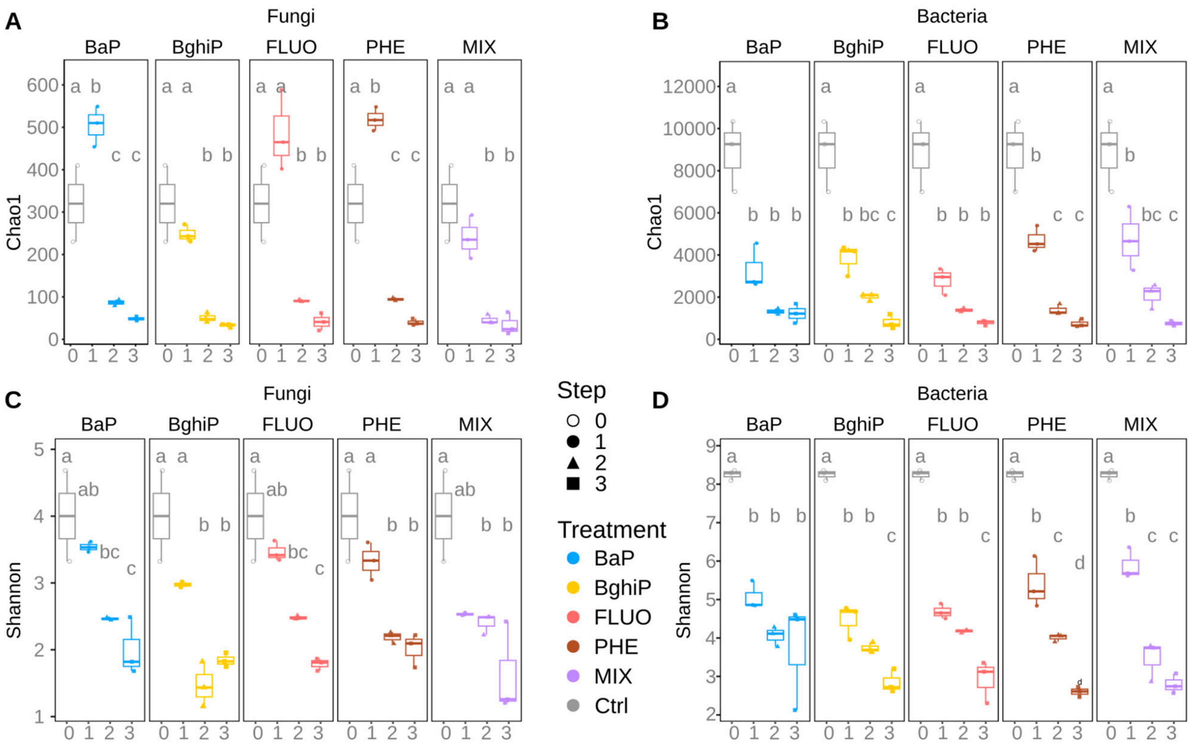

Figure S3. Barplots showing the relative abundance of the most represented fungal phyla, classes and orders.

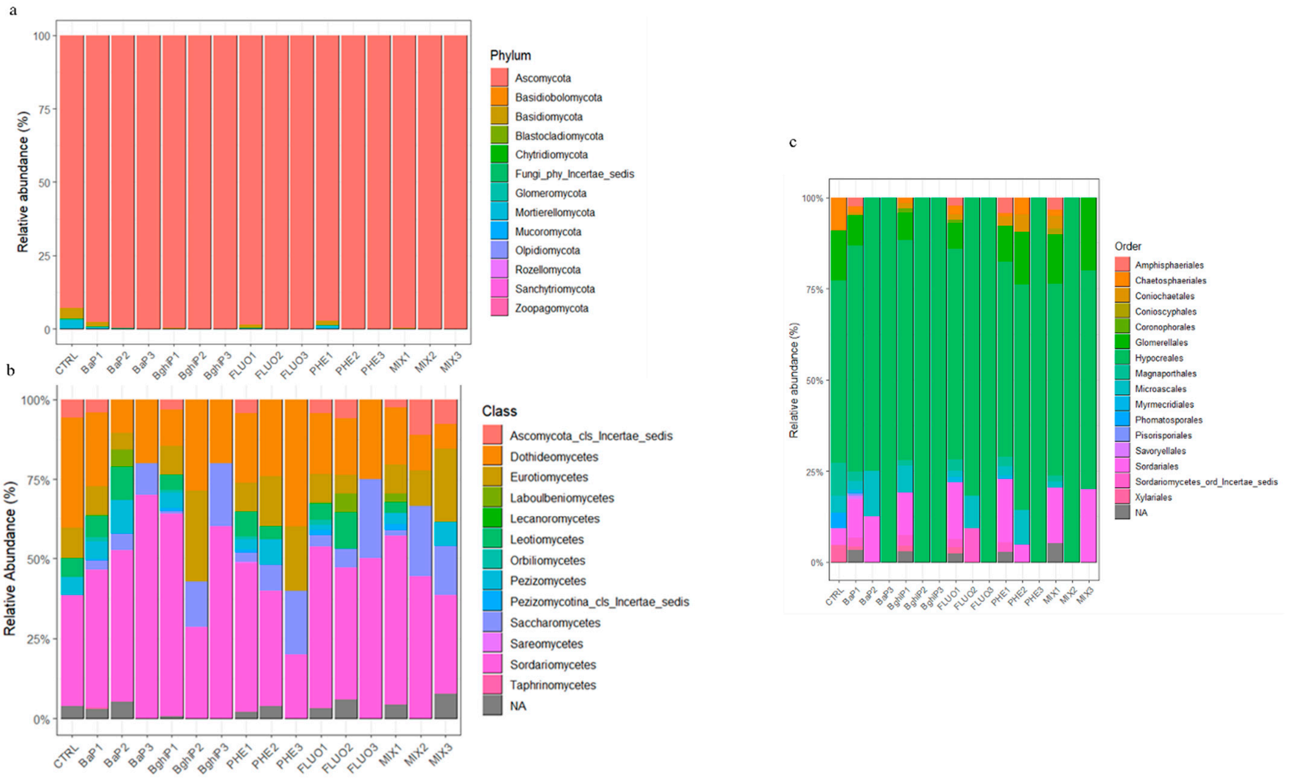

Figure S4. Concentration of PHE in percentage at the beginning (T0) and at the end (T7) of each enrichment step. Different lowercases indicate statistical significance (unpaired t-test; $p<0.05$)

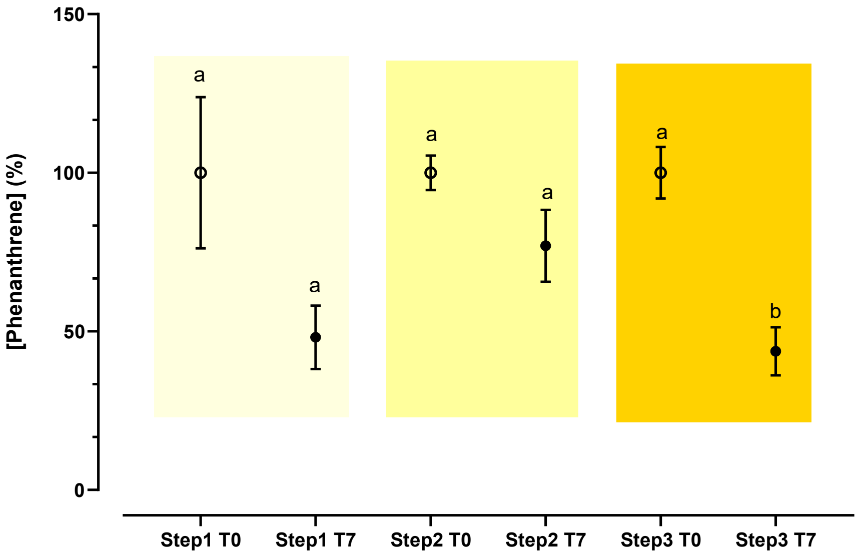

Table S1. alpha diversity measures of the sequenced samples. PHE=phenanthrene; FlUO=fluoranthene; BaP=benzo(a)pyrene; BGHI=benzo(g,h,i)perylene; MIX=Mix of the four PAHs. OBS.= Observed diversity.

		Fungi					Bacteria				
**PAH**	**Step**	**Sample_Id**	**Obs**	**Chao1**	**Shannon**	**Simpson**	**Sample_ Id**	**Obs**	**Chao1**	**Shannon**	**Simpson**
**PHE**	1	F1	301	404.00	2.99	0.87	B1	4496	4522.00	4.84	0.86
**PHE**	1	F2	332	418.22	3.56	0.92	B2	4299	4349.29	4.78	0.85
**PHE**	1	F3	291	376.02	3.29	0.90	B3	4153	4176.71	4.68	0.83
**FLUO**	1	F4	262	369.13	3.39	0.92	B4	2972	2992.72	3.95	0.77
**FLUO**	1	F5	293	461.00	3.31	0.91	B5	6238	6294.75	6.36	0.96
**FLUO**	1	F6	345	474.36	3.62	0.93	B6	4623	4653.85	5.62	0.92
**BaP**	1	F7	286	373.02	3.47	0.93	B7	3251	3274.89	5.68	0.95
**BaP**	1	F8	305	429.38	3.61	0.94	B8	1674	1680.63	4.08	0.92
**BaP**	1	F9	311	423.22	3.53	0.93	B9	1217	1220.19	4.05	0.90
**BGHI**	1	F10	144	225.67	2.92	0.90	B10	1259	1265.12	3.89	0.91
**BGHI**	1	F11	156	214.33	2.95	0.90	B11	1478	1485.35	4.19	0.92
**BGHI**	1	F12	151	201.17	3.03	0.90	B12	5366	5396.59	6.13	0.94
**MIX**	1	F13	165	214.50	2.57	0.82	B13	1298	1301.49	4.19	0.92
**MIX**	1	F14	170	227.42	2.50	0.82	B14	1365	1370.29	4.13	0.91
**MIX**	1	F15	151	177.40	2.53	0.82	B15	1305	1308.90	4.28	0.92
**PHE**	2	F16	82	82.83	2.21	0.78	B16	1181	1184.24	3.77	0.90
**PHE**	2	F17	77	84.86	2.09	0.76	B17	1456	1462.48	4.11	0.90
**PHE**	2	F18	82	88.11	2.24	0.79	B18	2095	2102.12	3.88	0.82
**FLUO**	2	F19	80	85.00	2.46	0.85	B19	1804	1811.59	3.63	0.87
**FLUO**	2	F20	76	77.67	2.49	0.85	B20	2096	2102.91	3.68	0.80
**FLUO**	2	F21	83	86.27	2.50	0.85	B21	2559	2565.33	3.79	0.87
**BaP**	2	F22	85	89.50	2.49	0.85	B22	1425	1431.63	2.86	0.75
**BaP**	2	F23	73	77.67	2.45	0.85	B23	4166	4201.66	5.21	0.88
**BaP**	2	F24	80	89.00	2.46	0.85	B24	2278	2285.72	3.73	0.87
**BGHI**	2	F25	45	45.75	1.42	0.49	B25	614	615.32	2.61	0.76
**BGHI**	2	F26	38	38.00	1.18	0.41	B26	969	970.57	2.73	0.78
**BGHI**	2	F27	60	60.86	1.84	0.62	B27	653	654.53	2.46	0.74
**MIX**	2	F28	39	39.33	2.49	0.88	B28	872	874.27	3.35	0.83
**MIX**	2	F29	57	64.50	2.23	0.79	B29	840	841.40	2.30	0.59
**MIX**	2	F30	39	39.00	2.50	0.88	B30	641	644.70	3.122	0.80
**PHE**	3	F31	32	32.00	1.72	0.67	B31	1679	1687.46	4.60	0.94
**PHE**	3	F32	36	36.00	2.13	0.79	B32	766	766.86	2.12	0.55
**PHE**	3	F33	47	47.60	2.23	0.82	B33	1214	1218.82	4.48	0.93
**FLUO**	3	F34	56	56.33	1.86	0.66	B34	2081	2091.81	4.50	0.92
**FLUO**	3	F35	38	38.25	1.68	0.64	B35	691	691.89	2.72	0.83
**FLUO**	3	F36	21	21	1.80	0.69	B36	1187	1188.89	3.20	0.88
**BaP**	3	F37	44	45.50	1.66	0.55	B37	535	536.26	2.62	0.81

**BaP**	3	F38	52	54.00	2.47	0.84	B38	874	874.85	2.74	0.83
**BaP**	3	F39	46	47.50	1.81	0.61	B39	734	735.68	3.076	0.88
**BGHI**	3	F40	34	35.00	1.85	0.74	B40	640	641.25	2.571	0.80
**BGHI**	3	F41	26	26.50	1.97	0.81	B41	2936	2953.70	4.65	0.87
**BGHI**	3	F42	29	29.00	1.75	0.71	B42	3319	3339.75	4.89	0.88
**MIX**	3	F43	22	22.75	1.29	0.62	B43	2605	2617.61	4.85	0.90
**MIX**	3	F44	59	81.75	2.40	0.87	B44	4531	4558.43	5.49	0.95
**MIX**	3	F45	14	14.00	1.21	0.60	B45	2708	2726.75	4.84	0.89
**SOIL**	0	FA	226	227.25	4.69	0.98	BA	9145	9258.19	8.28	0.99
**SOIL**	0	FB					BB	6925	7000.14	8.09	0.99
**SOIL**	0	FC	335	377.38	3.28	0.81	BC	10201	10332.01	8.35	0.99

Table S2. Taxonomy of the retrieved fungal isolates.

**Phylum**	**Class**	**Order**	**Family**	**Genus**	**Taxon**
Ascomycota	Dipodascomycetes	Dipodascales	Dipodascaceae	*Galactomyces*	*Galactomyces pseudocandidus*
	Dothideomycetes	Cladosporiales	Cladosporiaceae	*Cladosporium*	*Cladosporium allicinum*
					Cladosporium cladosporioides
					Cladosporium langeronii
					Cladosporium westerdijkia
	Euromycetes	Chaetothyriales	Herpotrichiellaceae	*Exophiala*	*Exophiala attenuata*
		Eurotiales	Aspergillaceae	*Aspergillus*	*Aspergillus fumigatus*
				Penicillium	*Penicillium chrysogenum*
					Penicillium crustosum
			Trichocomaceae	*Talaromyces*	*Talaromyces wortmannii*
	Sordariomycetes	Hypocreales	Nectriaceae	*Fusarium*	*Fusarium falciforme*
					Fusarium oxysporum
					Fusarium solani
				Paracremonium	*Paracremonium* sp.
			Hypocreales i.s.	*Stilbella*	*Stilbella aciculosa*
		Microascales	Microascaceae	*Scedosporium*	*Scedosporium apiospermum*
					Scedosporium dehogii
		Trichosphaeriales	Trichosphaeriaceae	*Gibellulopsis*	*Gibellulopsis nigrescens*
Basidiomycota	Agaricomycetes	Polyporales	Polyporaceae	*Trametes*	*Trametes versicolor*

Table S3. Fungal taxa retrieved from different conditions. For each taxa associated bacteria are reported. Equal upper case letters within the same taxon indicate more isolates of the same strain.

		Isolation Substrate					
**Fungal Taxon**	**Isolate Original Acronym**	**PHE**	**FLUO**	**BaP**	**BghiP**	**MIX**	**Associated Bacteria**
Aspergillus fumigatus	26		X				-
Aspergillus fumigatus	27		X				-
Aspergillus fumigatus	34		X				-
Cladosporium allicinum	58			X			-
Cladosporium cladosporioides	46		X				-
Cladosporium langeronii	14	X					-
Cladosporium westerdijkia	43		X				-
Exophiala attenuata [a]	56			X			-
Exophiala attenuata [a]	60			X			-
Exophiala attenuata	61			X			-
Exophiala attenuata [a]	63			X			-
Exophiala attenuata	33		X				*Chitinophaga arvensicola*
Exophiala attenuata	36		X				*Chitinophaga arvensicola*
Fusarium falciforme	99					X	-
Fusarium oxysporum	7	X					-
Fusarium oxysporum	49			X			-
Fusarium oxysporum	64			X			-
Fusarium oxysporum	95					X	-
Fusarium oxysporum	31		X				-
Fusarium solani	35		X				-
Fusarium solani [a]	44		X				-
Fusarium solani [b]	84				X		-
Fusarium solani [b]	100					X	-
Fusarium solani [a]	102					X	-
Galactomyces pseudocandidus [c]	1	X					-
Galactomyces pseudocandidus	3	X					-
Galactomyces pseudocandidus	4	X					*Bosea robiniae*
Galactomyces pseudocandidus [c]	5	X					-
Galactomyces pseudocandidus [c]	9	X					-
Galactomyces pseudocandidus [c]	10	X					-
Galactomyces pseudocandidus [c]	11	X					-
Galactomyces pseudocandidus [b]	12	X					-
Galactomyces pseudocandidus [g]	15	X					-

Galactomyces pseudocandidus	17	X					*Stenotrophomonas maltophilia*
Galactomyces pseudocandidus [c]	18	X					*Variovorax boronicumulans*
Galactomyces pseudocandidus [a]	19	X					-
Galactomyces pseudocandidus [a]	20	X					-
Galactomyces pseudocandidus [h]	21	X					-
Galactomyces pseudocandidus	24		X				*Chitinophaga ginsengisegetis*
Galactomyces pseudocandidus [b]	25		X				-
Galactomyces pseudocandidus [a]	29		X				-
Galactomyces pseudocandidus [g]	30		X				-
Galactomyces pseudocandidus [b]	32		X				*Bosea robiniae*
Galactomyces pseudocandidus [b]	38		X				*Bosea robiniae*
Galactomyces pseudocandidus [b]	41		X				-
Galactomyces pseudocandidus [b]	42		X				-
Galactomyces pseudocandidus [b]	45		X				*Pseudomonas* sp.
Galactomyces pseudocandidus [c]	47			X			*Bosea robiniae*
Galactomyces pseudocandidus [c]	48			X			*Chitinophaga ginsengisegetis*
Galactomyces pseudocandidus	50			X			-
Galactomyces pseudocandidus [h]	52			X			-
Galactomyces pseudocandidus [a]	55			X			-
Galactomyces pseudocandidus	59			X			*Bosea robiniae*
Galactomyces pseudocandidus [b]	65			X			-
Galactomyces pseudocandidus [e]	66				X		-
Galactomyces pseudocandidus [e]	67				X		-
Galactomyces pseudocandidus [d]	68				X		-
Galactomyces pseudocandidus [a]	69				X		*Stenotrophomonas* sp.
Galactomyces pseudocandidus [d]	71				X		-
Galactomyces pseudocandidus [f]	72				X		-
Galactomyces pseudocandidus [d]	74				X		-
Galactomyces pseudocandidus [a]	75				X		-
Galactomyces pseudocandidus [a]	76				X		Putative Consortium
Galactomyces pseudocandidus [a]	77				X		-
Galactomyces pseudocandidus [a]	78				X		Putative Consortium
Galactomyces pseudocandidus	80				X		-
Galactomyces pseudocandidus [a]	81				X		-
Galactomyces pseudocandidus [a]	82				X		*Stenotrophomonas* sp.
Galactomyces pseudocandidus [a]	83				X		-
Galactomyces pseudocandidus [a]	85				X		Putative Consortium
Galactomyces pseudocandidus	87					X	*Stenotrophomonas* sp.

Galactomyces pseudocandidus [d]	88					X	Putative Consortium
Galactomyces pseudocandidus	89					X	*Stenotrophomonas* sp.
Galactomyces pseudocandidus	90					X	Putative Consortium
Galactomyces pseudocandidus	92					X	Putative Consortium
Galactomyces pseudocandidus	93					X	*Stenotrophomonas* sp.
Galactomyces pseudocandidus	94					X	Putative Consortium
Galactomyces pseudocandidus [f]	96					X	*Stenotrophomonas* sp.
Galactomyces pseudocandidus	97					X	*Stenotrophomonas maltophilia*
Galactomyces pseudocandidus [a]	101					X	*Stenotrophomonas* sp.
Galactomyces pseudocandidus [a]	103					X	*Stenotrophomonas indicatrix*
Galactomyces pseudocandidus [a]	104					X	*Stenotrophomonas* sp.
Galactomyces pseudocandidus [a]	57			X			-
Gibellulopsis nigrescens	53			X			-
Gibellulopsis nigrescens	62			X			-
Paracremonium sp.	22		X				-
Paracremonium sp.	79				X		-
Penicillium chrysogenum	2	X					-
Penicillium crustosum	23		X				-
Scedosporium apiospermum	70				X		-
Scedosporium dehogii	86					X	*Stenotrophomonas indicatrix*
Scedosporium dehogii	91					X	-
Stilbella aciculosa [a]	6	X					-
Stilbella aciculosa [a]	8						-
Stilbella aciculosa [a]	13	X					-
Stilbella aciculosa [a]	16	X					-
Stilbella aciculosa	28		X				*Chitinophaga arvensicola*
Stilbella aciculosa	37		X				-
Stilbella aciculosa	51			X			Putative Consortium
Stilbella aciculosa	54			X			*Chitinophaga ginsengisegetis*
Talaromyces wortmannii	39		X				-
Trametes versicolor	40		X				*Bosea robiniae*

Table S4. Correlation between Biosurfactant production, bacterial presence and fungal growth

**Biosurfactants production vs Fungal growth**	**Bacterial presence vs Fungal growth**
**PHE**: $\rho = -0.239$, $p = 0.480$, $n = 11$	**PHE**: $\rho = -0.087$, $p = 0.800$, $n = 11$
**FLUO**: $\rho = -0.199$, $p = 0.557$, $n = 11$	**FLUO**: $\rho = -0.313$, $p = 0.349$, $n = 11$
**BaP**: $\rho = +0.388$, $p = 0.239$, $n = 11$	**BaP**: $\rho = -0.093$, $p = 0.786$, $n = 11$
**BghiP**: $\rho = +0.190$, $p = 0.576$, $n = 11$	**BghiP**: $\rho = -0.021$, $p = 0.950$, $n = 11$
**MIX**: $\rho = +0.101$, $p = 0.768$, $n = 11$	**MIX**: $\rho = +0.035$, $p = 0.919$, $n = 11$
